# MicroRNA and mRNA expression profiling analysis revealed the regulation of plant height in *Gossypium hirsutum*

**DOI:** 10.1186/s12864-015-2071-6

**Published:** 2015-10-30

**Authors:** Wenyan An, Wenfang Gong, Shoupu He, Zhaoe Pan, Junling Sun, Xiongming Du

**Affiliations:** State Key Laboratory of Cotton Biology/Institute of Cotton Research, Chinese Academy of Agricultural Sciences, Anyang, 455000 Henan China; College of Life Science and Technology, Huazhong Agricultural University, Wuhan, 430072 Hubei China

**Keywords:** *Gossypium hirsutum*, microRNA, mRNA, Dwarf mutant, Tall-culm mutant

## Abstract

**Background:**

Dwarf cottons are more resistant to damage from wind and rain and associated with stable, increased yields, and also desirable source for breeding the machine harvest varieties. In an effort to uncover the transcripts and miRNA networks involved in plant height, the transcriptome and small RNA sequencing were performed based on dwarf mutant Ari1327 (A1), tall-culm mutant Ari3697 (A3) and wild type Ari971 (A9) in *Gossypium hirsutum*.

**Methods:**

The stem apexes of wild-type upland cotton (Ari971) and its dwarf mutant (Ari1327) and tall-culm mutant (Ari3697) at the fifth true leaf stage were extracted for RNA, respectively. Transcriptome and small RNA libraries were constructed and subjected to next generation sequencing.

**Results:**

The transcriptome sequencing analysis showed that the enriched pathways of top 3 differentially expressed genes (DEGs) were categorized as carotenoid biosynthesis, plant-pathogen interaction and plant hormone signal transduction in both A1–A9 and A3–A9. The ABA and IAA related factors were differentially expressed in the mutants. Importantly, we found the lower expressed *SAUR* and elevated expressed *GH3*, and ABA related genes such as *NCED* and *PP2C* maybe relate to reduced growth of the plant height in Ari1327 which was consistent with the higher auxin and ABA content in this mutant. Furthermore, miRNA160 targeted to the auxin response factor (*ARF*) and miRNA166 (gma-miR166u and gma-miR166h-3p) targeted to ABA responsive element binding factor were related to the mutation in cotton. We have noticed that the cell growth related factors (*smg7* targeted by gra-miR482 and 6 novel miRNAs and *pectate-lyases* targeted by osa-miR159f), the redox reactions related factors (*Cytochrome P450* targeted by miR172) and *MYB* genes targeted by miR828, miR858 and miR159 were also involved in plant height of the cotton mutants. A total of 226 conserved miRNAs representing 32 known miRNA families were obtained, and 38 novel miRNAs corresponding to 23 unique RNA sequences were identified. Total 531 targets for 211 conserved miRNAs were obtained. Using PAREsnip, 27 and 29 miRNA/target conserved interactions were validated in A1–A9 and A3–A9, respectively. Furthermore, miRNA160, miRNA858 and miRNA172 were validated to be up-regulated in A1–A9 but down-regulated in A3–A9, whereas miRNA159 showed the opposite regulation.

**Conclusions:**

This comprehensive interaction of the transcriptome and miRNA at tall-culm and dwarf mutant led to the discovery of regulatory mechanisms in plant height. It also provides the basis for in depth analyses of dwarf mutant genes for further breeding of dwarf cotton.

**Electronic supplementary material:**

The online version of this article (doi:10.1186/s12864-015-2071-6) contains supplementary material, which is available to authorized users.

## Background

Cotton is one of the most important economic crops and provides the majority of natural fiber materials worldwide. Plant height is an important trait in cotton, and dwarf cottons are more resistant to damage from wind and rain and associated with stable, increased yields. Because of their agronomic importance, the different types of dwarf mutants have been isolated from rice [1], *Arabidopsis* [2], and wheat [3].

The development of dwarf cultivars has played a significant role in plant breeding, growth and development. However, fewer studies have been carried out in cotton species. Hutchinson and Ghose [4] found a crinkled dwarf in upland cotton, which showed a normal phenotype during the seedling stage and a crinkled dwarf phenotype at the fourth- or fifth-leaf stage. Genetic analysis showed the mutant to be controlled by a completely recessive gene. A novel super-dwarf mutant, named AS98, was discovered from an interspecific hybrid in 1998. Compared to the normal line LHF10W99, plant height and internode length were significantly shorter in AS98, but it had only slightly (3–5 %) fewer internodes [5]. The trait was controlled by a single incomplete-dominant gene, and exogenous gibberellins 3 (GA3) could restore plant height in AS98. In addition, a cotton mutant, *pag1*, exhibited dwarfism due to significant inhibition of cell elongation and expansion, and brassinolide (BL) treatment rescued its growth and fiber elongation [6] These results indicate that most dwarf mutants are controlled by recessive genes, and involve very few dominant genes, and plant hormones play significant roles in plant height decision.

In plants, miRNAs are small endogenous RNAs and play negative regulatory functions at the post-transcriptional level by repressing gene translation or degrading target mRNAs [7]. MiRNAs display near-perfect complementarities to their target mRNAs and interfere with target gene expression by mRNA cleavage, which occurs at the 10th and 11th positions of miRNAs, or by inhibition of translation in plants [8]. The path in which miRNAs work is determined by the sequence complementarity of miRNAs to their target mRNAs. If the miRNA has a perfect sequence complementarity to the mRNA, the mRNA is targeted for cleavage; or, protein translation is inhibited [9, 10]. Most miRNAs are high conserved among plant species [11]. There also exist non-conserved or species-specific miRNAs which often expressed at very low levels, and many are not found in small-scale sequencing projects. Recently, using next-generation sequencing technology, many new non-conserved miRNAs with low abundance could be identified [12].

Many miRNAs have been identified using experimental and/or bioinformatics approaches in various plant species [13, 14], the number of identified tissue-, species-, and developmental stage-specific miRNAs is still limited because of their low accumulations levels. The advent of high-throughput sequencing technologies has allowed the mining of these specific low-abundance miRNAs. To date, 321, 337 and 713 mature miRNAs have been uploaded to the miRBase database from Zea mays, *Arabidopsis thaliana* and rice, respectively [15, 16]. However, only 80 mature miRNAs from *G. hirsutum* had been annotated in the miRBase database (release 20). Due to the limited numbers of *G. hirsutum* EST sequences in the public NCBI database, data collected from transcriptome sequencing was used as reference sequences, which provide more valuable information for prediction of conserved miRNAs.

Numerous studies have indicated that miRNAs have proven to be involved in many functional processes such as leaf development, shoot and root development, floral development, hormone response and stress adaption [17–20]. MiR160 was essential for root cap formation and proper plant development [21–23]. MiR159 was shown to be a phytohormonally regulated homeostatic modulator of *GAMYB* activity and *GAMYB*-depended developmental processes, such as flowering time and anther development [24]. The expression assay of miRNAs under Cd stress in *M. truncatula* found that miR393, miR171, miR319, and miR529 were up-regulated, whereas miR166 and miR398 were down-regulated [25].

To investigate the relationships between miRNAs and cotton plant height, three miRNA libraries and three cDNA libraries were constructed using the stem apex of three samples [dwarf mutant Ari1327 (A1), tall-culm mutant Ari3697 (A3) and wild type Ari971 (A9) upland cottons], and sequenced by Solexa technology. Then miRNAs and their targets were analyzed. This is the first report that analyzes both dwarf mutant and tall-culm mutant under mRNA and miRNAs levels using high-throughput sequencing, and it will help us to investigate the dwarf mutant gene for further cotton dwarf breeding.

## Results

### Differences of plant height of the mutants

**Fig. 1:**
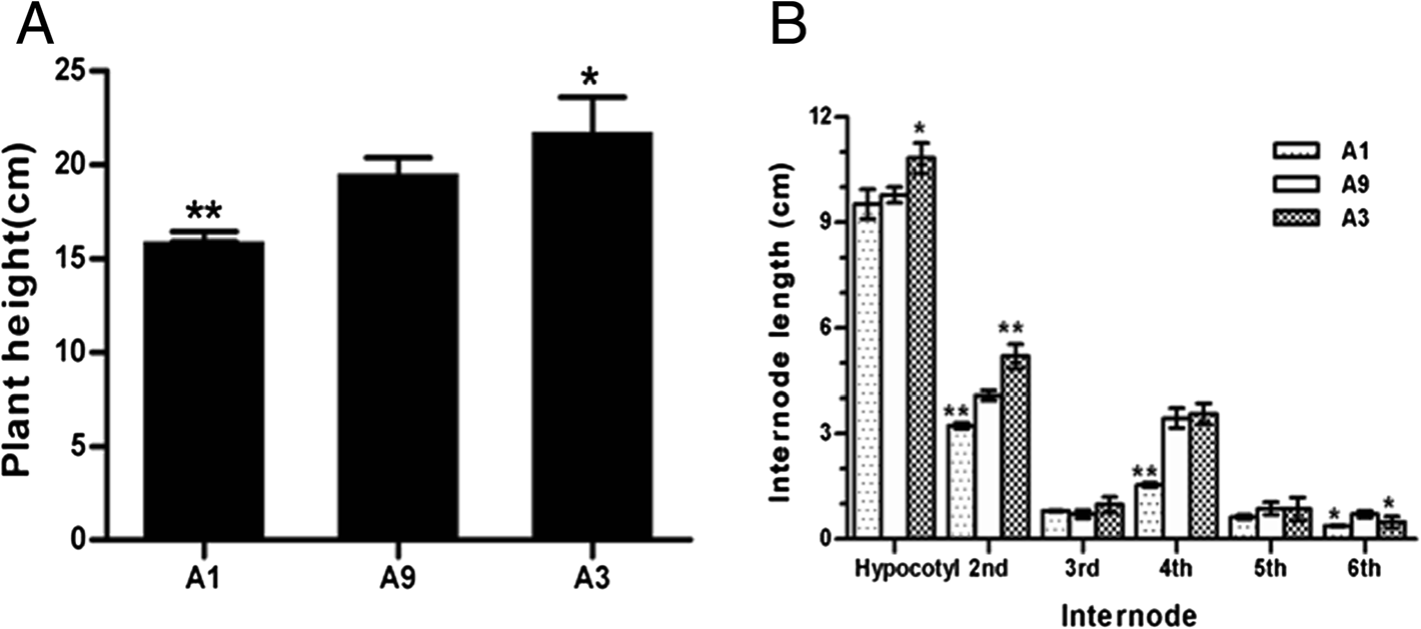
Morphological phenotypes of Ari971, Ari1327 and Ari3697 at the fifth true leaf stage. **a** The plant height of dwarf mutant (A1), wild type (A9) and tall-culm mutant (A3). **b** Lengths of individual internodes in the A1, A9, and A3 plants. Data of each sample was the means from 60 plants. Error bars represent SD. value with * represented significant difference at *P* < 0.01 while ** represented highly significant difference at *P* < 0.001

At the fifth true leaf stage, significant differences of plant height could be observed. In dwarf mutant A1, the plant height was 82 % of that in wild type A9 (highly significant, *p* < 0.01, student’s *t* test), while the tall-culm mutant A3 was 11 % taller than wild type (significant, *p* < 0.05, student’s *t* test) (Fig. 1a). The hypocotyls of A1 and A9 were slightly different; but it was different significantly between A3 and A9. The length of the second and the fourth internodes of A1 were 55 and 21 % shorter than that of A9, respectively. The highly significant difference between A3 and A9 were the second internode with 27 % longer (Fig. 1b). However, the number of internodes of A1, A3, and A9 was no difference, indicating that the length of internodes causes dwarf mutant.

### Overview transcriptome sequencing

cDNA libraries were constructed from stem apex collected from A1, A9 and A3, and paired-end sequenced by Solexa SBS. As shown in Table 1, a total of 60,819,352 raw reads were obtained from these three libraries. After filtering low quality reads and removing sequences with N, 17,142,420 (valid ratio 88.43 %) clean reads from A1, 16,728,306 (valid ratio 86.04 %) from A3 and 18,712,824 (85.10 %) from A9 were obtained, respectively. Clean reads of all the three libraries were *de novo* assembled by Trinity. A total of 156,848 transcripts after removing repeats were obtained, and 70,877 unigenes were obtained.

**Table 1:** Data set summary of sequencing of 3 small RNA and transcriptome libraries

	Category	A1	A3	A9
Small RNA data	Raw reads	13,254,110	11,148,419	10,195,723
	Clean reads	6,533,407	5,815,756	4,613,890
	Unique reads	816,133	550,764	331,333
	Match tRNAdb/SILVA Rrna/NONCODE v3.0	49,006	52,345	65,095
	Known miRNAs	513	495	389
	Unannotated	766,614	497,924	265,849
Transcriptome	Raw reads	19,386,166	19,442,786	21,990,400
	Valid reads	17,142,420	16,728,306	18,712,824
	Valid ratio	88.43 %	86.04 %	85.10 %
	All transcript	156,848		
	All unigene	70,877		

Differential expression analysis showed that total 33,151 unigenes showed differentially expressed significantly by comparing A1 and A9 libraries (False discovery rate ≤0.001, │log2Ratio│ ≥ 1), 15,194 (45.83 %) of them up-regulated, while 17,957 (54.17 %) of them down-regulated. In addition, compared A3 to A9, 14,325(46.73 %) unigenes were up-regulated, while 16,331 (53.27 %) unigenes were down-regulated.

Gene Ontology (GO) analysis result showed that the top 5 differentially expressed genes (DEGs) enriched Gene Ontology terms of both A1–A9 and A3–A9 included RNA-directed DNA polymerase activity, RNA-dependent DNA replication, DNA integration, defense response, and apoptotic process (Table 2).

**Table 2:** GO and KEGG analysis of DEGs

	DEGs	GO	*P*-value	KEGG	*P*-value
A1–A9	33,151	RNA-directed DNA polymerase activity	4.70E-190	Carotenoid biosynthesis	1.24E-26
		RNA-dependent DNA replication	1.58E-158	Plant hormone signal transduction	8.60E-21
		DNA integration	2.92E-124	Plant-pathogen interaction	4.95E-19
		Defense response	2.55E-80	Arachidonic acid metabolism	3.68E-14
		Apoptotic process	2.81E-75	Pertussis	1.71E-13
A3–A9	30,656	RNA-directed DNA polymerase activity	3.15E-228	Carotenoid biosynthesis	1.69E-27
		RNA-dependent DNA replication	1.16E-195	Phenylpropanoid biosynthesis	2.08E-15
		DNA integration	7.44E-147	Plant-pathogen interaction	2.08E-14
		Defense response	4.48E-71	Plant hormone signal transduction	2.07E-13
		Apoptotic process	1.99E-65	Complement and coagulation cascades	2.40E-10

The Kyoto Encyclopedia of Genes and Genomes (KEGG) pathway analysis showed the top 5 DEGs enriched pathways of A1–A9 and A3–A9, and 3 of them were categorized as carotenoid biosynthesis, plant-pathogen interaction, and plant hormone signal transduction (Table 2). In the carotenoid biosynthesis pathway between A1 and A9, as the most induced gene, (+)-abscisic acid 8′-hydroxylase changed by 5.64 fold, while *NCED*, as the most down-regulated gene, changed by −4.15 fold. However, between A3 and A9,(+)-abscisic acid 8′-hydroxylase and *NCED* changed by 3.97 and 3.5 fold respectively, while abscisic-aldehyde oxidase (*AAO3*), xanthoxin dehydrogenase (*ABA2*) changed by −2 fold and −1.34 fold, respectively. In the plant-pathogen interaction pathway, *EFR* and *MIN7* were the most two down-regulated genes, while *CML*, *WRKY33* and *RBOH* were the most three induced genes between A1 and A9 genotype. Furthermore, *CML* and *CNGF* were the most two induced genes, while *RPM1* and *RBOH* were the most two down-regulated genes between A3 and A9. In the plant hormone signal transduction pathway, *SAUR*, *EBF1_2* and *PR1* were the most down-regulated three genes, while *TCH4*, *ERF1* and *DELLA* were the most three induced genes between A1 and A9. Furthermore, *GH3, SAUR* and *PP2C* were the most three down-regulated genes, while *TCH4*, *SNRK2*, and *CTR1* were the most three induced genes between A3 and A9.

### Identification and expression analyses of known miRNAs in the mutants

**Fig. 2:**
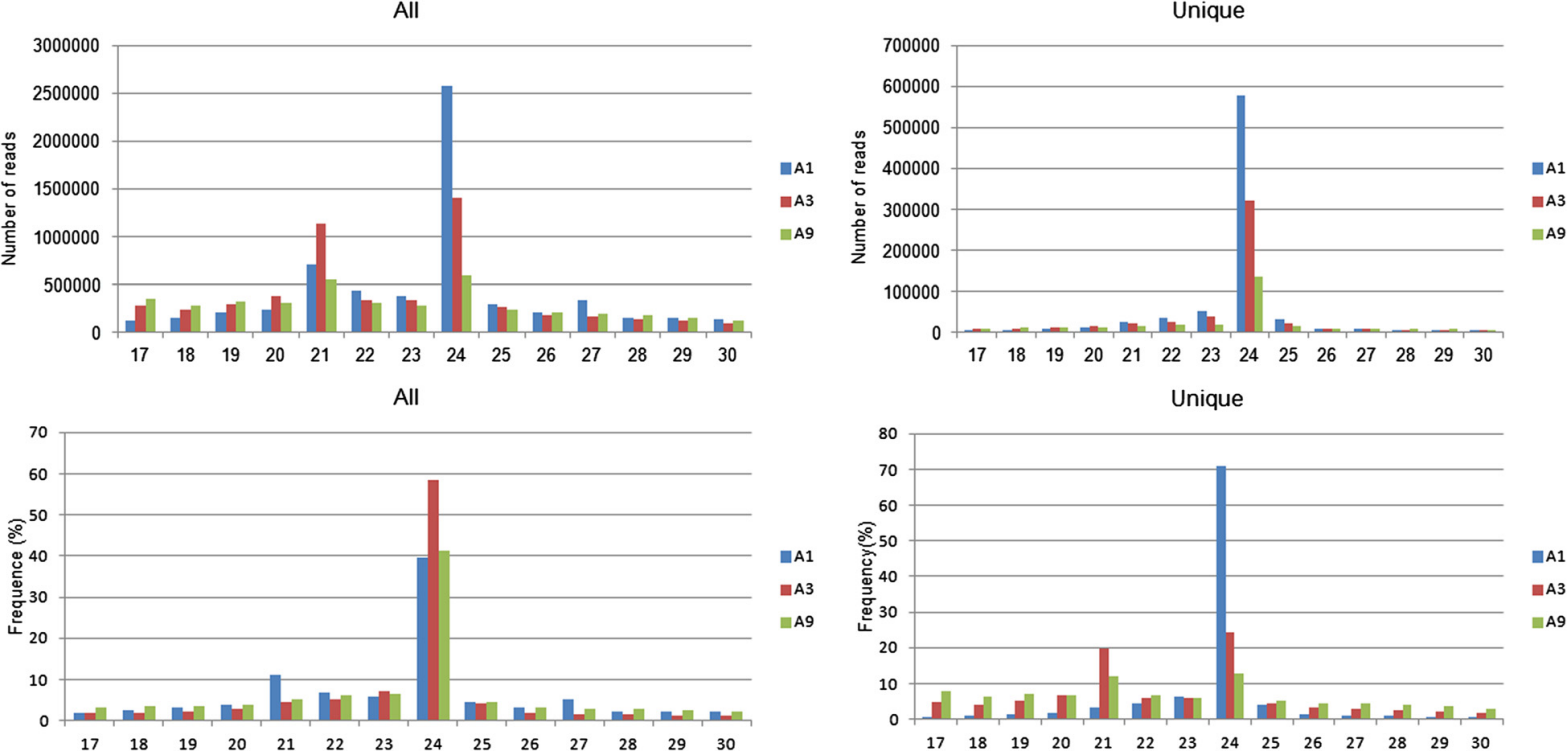
Total small RNA length distribution

Three small RNA libraries were constructed using total RNA obtained from stem apex of A1, A9 and A3, and sequenced. And 13,254,110, 11,148,419 and 10,195,723 raw reads from A1, A3 and A9 libraries were obtained, respectively, ranging from 17 to 35 nucleotides in length. As seen in Fig. 2, the highest abundant nucleotides in length was 24 nt followed by 21 nt. After removing adaptor contaminations and low quality reads, a total of 6,533,407 clean reads from A1 (representing 816,133 unique sequences), 5,815,756 from A3 (representing 550,764 unique sequences) and 4,613,890 from A9 (representing 331,333 unique sequences) were obtained. In order to remove rRNA, tRNA, snRNA and snoRNA (Additional file 1), all clean reads of three libraries were analyzed by blast against tRNAdb, SILVA rRNA and NONCODE v3.0 database. These small RNAs accounted for 49,006 unique reads in A1, 52,345 in A3 and 65,095 in A9, respectively (Table 1).

In total, 226 conserved miRNAs representing 32 known miRNA families were obtained (Table 3). The distribution of miRNAs in different miRNA families were shown as Additional file 2. Identified miRNAs belonging to miR159, miR166 and miR171 were the most abundant. Among these miRNAs, 198 from A1, 181 from A3 and 178 from A9 were perfectly matched to known miRNAs of other plant species, respectively. Of these known conserved miRNAs, 20 were identified as known cotton miRNAs, including eight cotton-specific miRNAs (miR479, miR2949b, miR2949a-5p, miR2949a-3p, miR482b, miR482a, miR2948-5p, and miR3476-5p), representing 15 miRNA families.

**Table 3:** Known miRNA families identified by similarity

Family	RPM			Log2	
	A1	A3	A9	A1–A9	A3–A9
miR156	1,157.81	278.61	1,038.36	0.16	−1.90
miR159	509,710.98	774,531.13	312,869.01	0.70	1.31
miR160	2,481.01	98.40	471.62	2.40	−2.26
miR162	2,081.68	169.15	499.36	2.06	−1.56
miR164	1,547.68	97.29	582.59	1.41	−2.58
miR166	404,503.62	214,922.06	654,186.53	−0.69	−1.61
miR167	6,261.59	208.96	820.38	2.93	−1.97
miR168	4,430.37	192.37	1,272.19	1.80	−2.73
miR169	18.90	5.53	0.01	10.88	9.11
miR171	5,106.15	498.62	1,803.26	1.50	−1.85
miR172	4,361.85	89.55	475.58	3.20	−2.41
miR2111	82.70	12.16	23.78	1.80	−0.97
miR2949	3,350.54	227.75	535.03	2.65	−1.23
miR2950	163.04	13.27	55.48	1.56	−2.06
miR319	330.02	733.11	541.29	−0.71	0.44
miR3630	134.68	49.75	317.06	−1.24	−2.67
miR390	5,198.30	313.98	1,894.41	1.46	−2.59
miR393	111.05	9.95	7.93	3.81	0.33
miR394	2,696.03	178.00	669.78	2.01	−1.91
miR395	340.25	138.20	233.83	0.54	−0.76
miR396	4,184.63	97.29	550.89	2.93	−2.50
miR399	6,025.31	371.48	1,042.32	2.53	−1.49
miR403	311.90	13.27	39.63	2.98	−1.58
miR408	11.81	125.93	41.61	−1.82	1.60
miR477	106.33	0.01	11.89	3.16	−10.22
miR482	23,931.10	3,301.27	9,313.53	1.36	−1.50
miR530	141.77	2.21	27.74	2.35	−3.65
miR535	510.38	186.84	772.83	−0.60	−2.05
miR827	5,647.25	1,003.87	2,853.51	0.98	−1.51
miR828	37.81	0.01	0.01	11.88	0.00
miR858	2.36	2.21	0.01	7.88	7.79
NA	5,021.09	2,127.81	7,048.56	−0.49	−1.73

RPM: Reads per million, 0 in A1, A3 or A9 was nomorlized as 0.01 to facilitate calculation; NA, unknown miRNA family

The miRNA expression level in mutant libraries and wild type library were compared. Total 14 miRNAs corresponding to 12 families were identified as being differentially expressed by comparing these three non-redundant libraries (*p* ≤ 0.05, │log_2_Ratio│ ≥ 1). And all the 11 differentially expressed miRNAs in cotton including ghr-miR162a, ghr-miR164a, ghr-miR167a, ghr-miR2949b, ghr-miR2949a-5p, ghr-miR390a, ghr-miR394a, ghr-miR396a, ghr-miR399d, ghr-miR482a and ghr-miR827 were up regulated in dwarf mutant (A1) but down regulated in tall-culm mutant (A3) compared with the wild type (A9), and three miRNA, ghr-miR479, ghr-miR2949a-3p and ghr-miR393b-5p were highly expressed in the dwarf mutant (A1) compared with that in the tall-culm mutant (A3) (Table 4).

**Table 4:** Known *G. hirsutum* miRNAs identified in each library

miRNA family	miRNA	miRNA sequence	RPM			Log_2_	
			A1	A9	A3	A1–A9	A3–A9
miR156	ghr-miR156a	UGACAGAAGAGAGUGAGCAC	19.85	38.84	9.29	−0.97	−2.06
miR162	ghr-miR162a	UCGAUAAACCUCUGCAUCCAG	1,830.80	364.61	126.20	2.33	−1.53
miR164	ghr-miR164a	UGGAGAAGCAGGGCACGUGCA	937.47	261.57	37.59	1.84	−2.80
miR166	ghr-miR166a	UCGGACCAGGCUUCAUUCCCC	143,209.00	199,713.00	57,708.00	−0.48	−1.79
miR167	ghr-miR167a	UGAAGCUGCCAGCAUGAUCUA	127.99	47.78	12.16	1.42	−1.97
miR171	ghr-miR479	CGUGAUAUUGGUUCGGCUCAUC	4.73	0.01	0.01	8.88	0.00
miR2949	ghr-miR2949b	UCUUUUGAACUGGAUUUGCCGA	14.18	5.94	1.11	1.25	−2.43
	ghr-miR2949a-5p	ACUUUUGAACUGGAUUUGCCGA	3,331.60	529.09	226.60	2.65	−1.22
	ghr-miR2949a-3p	UGCAAAUCCAGUCAAAAGUUA	4.73	0.01	0.01	8.88	0.00
miR390	ghr-miR390a	AAGCUCAGGAGGGAUAGCGCC	5,177.80	1,886.50	314.00	1.46	−2.59
miR393	ghr-miR393b-5p	UCCAAAGGGAUCGCAUUGAUCU	13.71	1.59	1.99	3.11	0.33
miR394	ghr-miR394a	UUGGCAUUCUGUCCACCUCC	1,387.00	340.84	95.08	2.02	−1.84
miR396	ghr-miR396a	UUCCACAGCUUUCUUGAACUG	1,723.00	131.18	35.93	3.72	−1.87
miR399	ghr-miR399d	UGCCAAAGGAGAUUUGCCCCG	326.08	35.67	17.69	3.19	−1.01
	ghr-miR399a	UGCCAAAGGAGAUUUGCCCUG	127.59	0.01	9.95	13.64	9.96
miR482	ghr-miR482b	UCUUGCCUACUCCACCCAUGCC	3,962.50	3,436.10	621.30	0.21	−2.47
	ghr-miR482a	UCUUUCCUACUCCUCCCAUACC	5,487.80	1,597.20	556.10	1.78	−1.52
	ghr-miR2948-5p	UGUGGGAGAGUUGGGCAAGAAU	1,984.80	499.36	1,00	1.99	1.00
miR827	ghr-miR827	UUAGAUGACCAUCAACAAACA	5,536.26	2,675.20	981.00	1.05	−1.45
miR3476	ghr-miR3476-5p	UGAACUGGGUUUGUUGGCUGC	2,384.10	2,096.50	1.442.00	0.19	−0.54

**Fig. 3:**
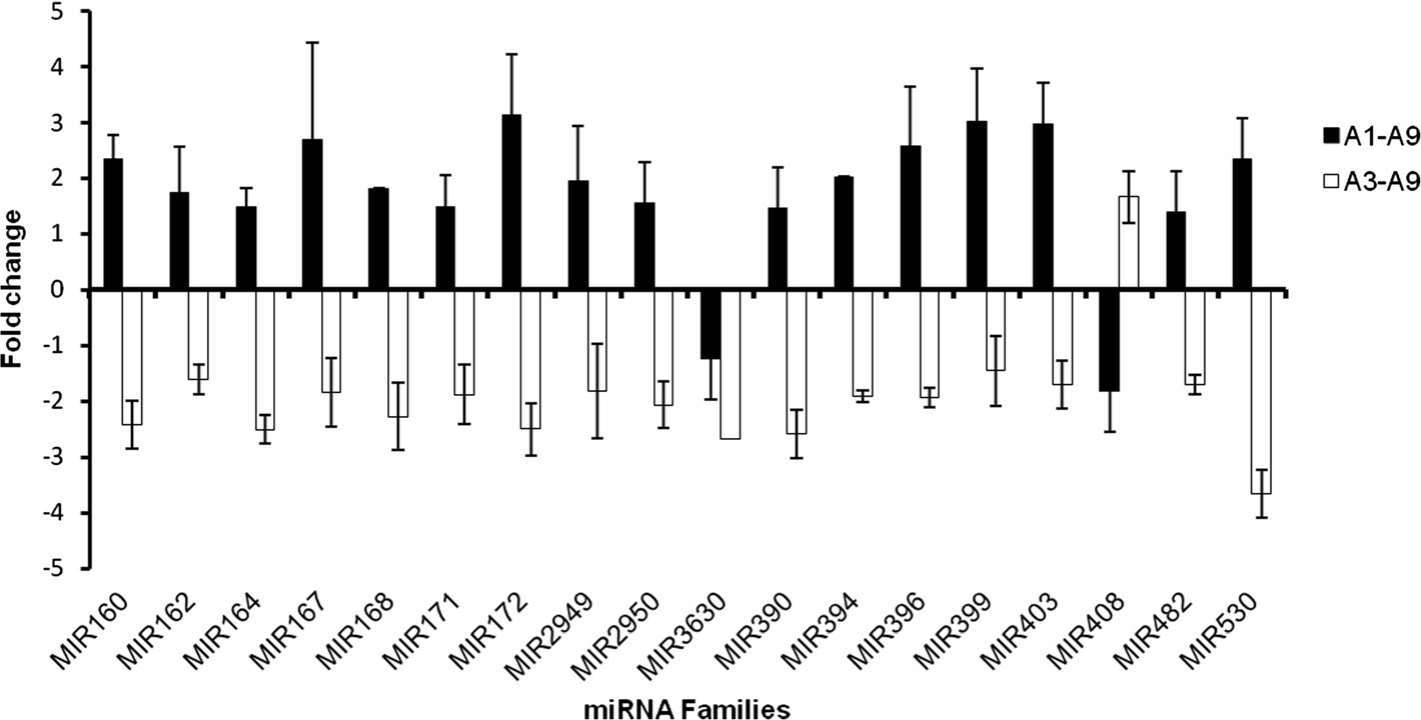
Differentially expressed known miRNA families of mutants and wild type. All the fold change differences of miRNA between A1–A9 and A3–A9 were significant at the 0.01 probability level

Among all the conserved miRNA families, 18 families were differentially expressed in A1–A9 and A3–A9. Of these, 16 miRNA families including miR160,miR162, miR164, miR167, miR168, miR171, miR172, miR2949, miR2950, miR390, miR394, miR396, miR399, miR403, miR482, miR530 were up regulated in dwarf mutant (A1) but down regulated in tall-culm mutant (A3) compared with the wild type (A9). On the contrary, miR3630 family was down regulated in both A1–A9 and A3–A9 and miR408 family was down regulated in A1 but up regulated in A3 compared with A9 (Fig. 3).

Twenty nine precursors were identified using miRDeep 2 (Additional file 3), and 14 miRNA*s (the complementary strands of functional mature miRNAs) were also detected for the knowing miRNAs with precursors. miRNA* sequences are rarely detected via conventional sequencing because of their rapid degradation in cells. The detection of miRNA* represented further evidence for the existence of mature miRNAs.

### Prediction of novel miRNAs

In total, 38 novel miRNAs corresponding to 23 unique RNA sequences were identified. Among these sequences, 24 nt were the most abundant fractions, which was the same as the length distribution of conserved small RNAs detected in this study. The length of the novel miRNA precursors varied from 70 to 101 nt with an average of 88 nt, which was consist with precious study that a majority of cotton miRNA precursor have 60–110 nucleotides [26]. The average minimum free energy (MFE) was −38.3 kcal mol^−1^, with a range of −81.9 kcal mol^−1^ to −20.6 kcal mol^−1^ (Additional file 4).

### Potential targets of conserved and putative novel miRNAs

**Fig. 4:**
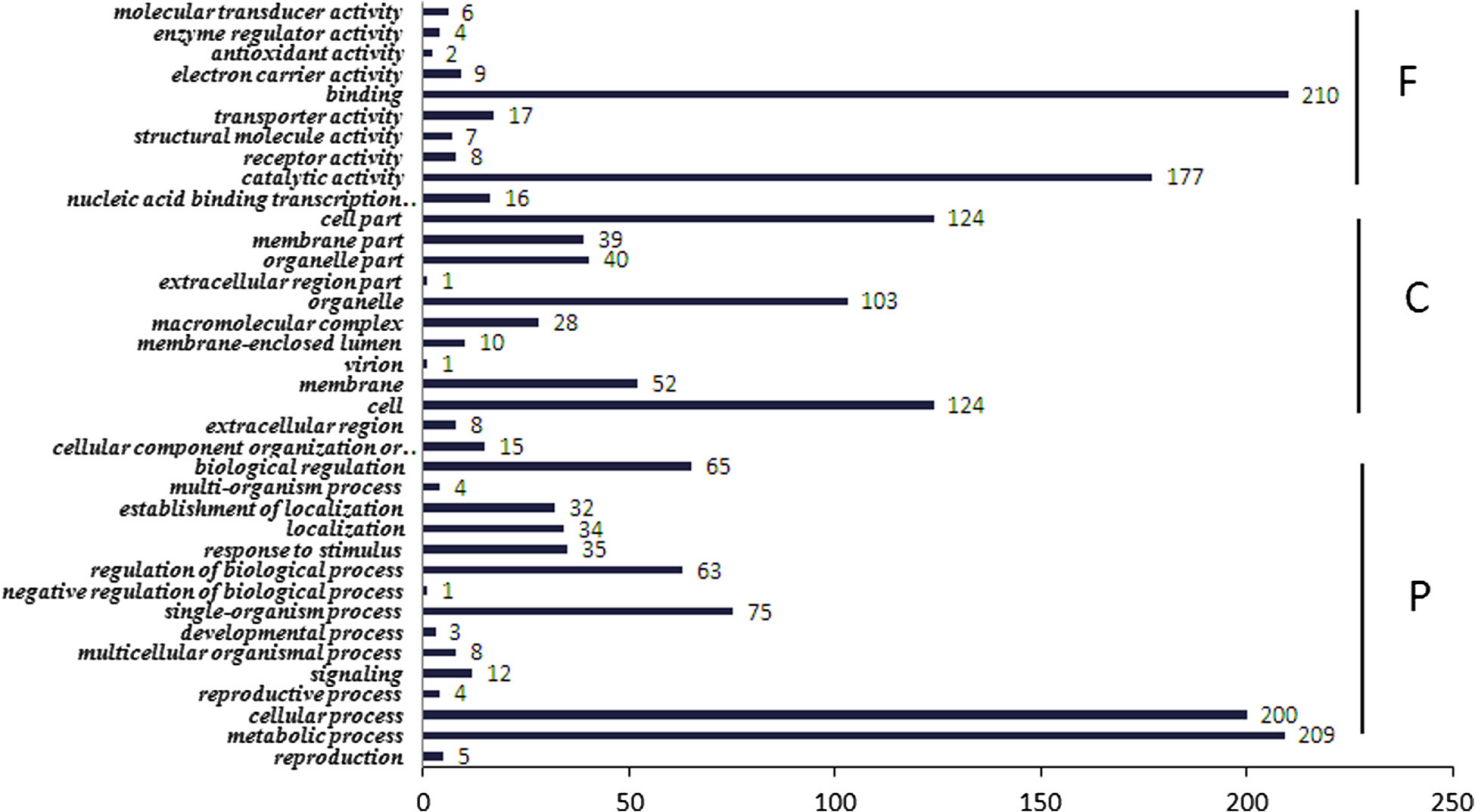
GO classification of conserved miRNA targets. F: molecular function, C: cellular component, P: biological process

**Fig. 5:**
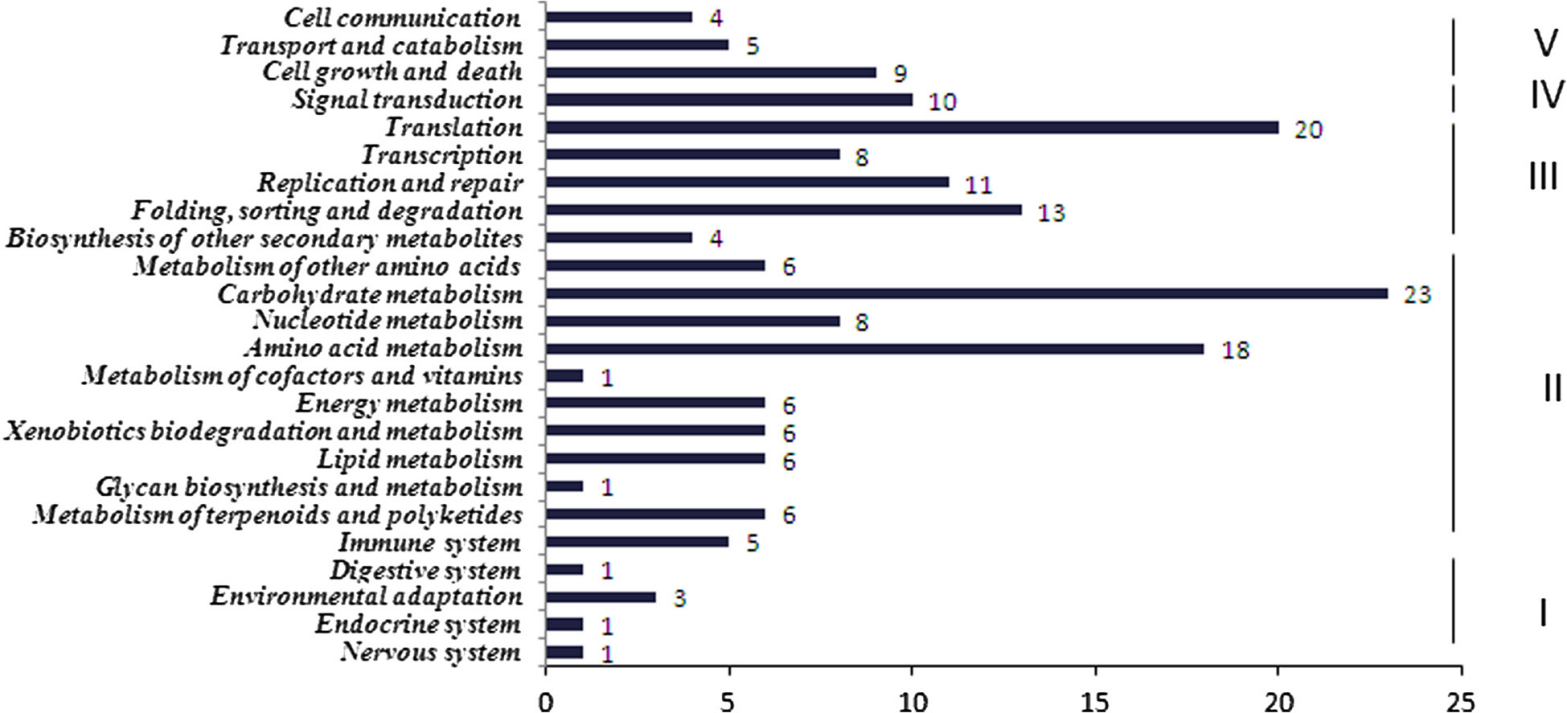
KEGG pathways of conserved miRNA targets. I: organism systems, II: metabolism, III: genetic information processing, IV: environmental information processing, V: cellular processes

A total of 531 targets for 211 conserved miRNAs were obtained and assigned to 436 GO functional classification and 116 KEGG pathways (Figs. 4 and 5). Based on the GO functional classification, most of the targets of all miRNAs correlated to binding, metabolic process, cellular process and catalytic activity. In the KEGG classification, carbohydrate metabolism enriched the most target genes, following with translation and amino acid metabolism. Ninety-seven miRNAs with 153 annotated targets different expressed between dwarf mutant (A1) and its wild type (A9). Among them, 74 miRNAs were up-regulated but 23 miRNAs were down-regulated. The Fold Change (log_2_Ratio) of gma-miR166u, vvi-miR396b, and aly-miR165a-3p was more than 3 with 4.60, 4.25 and 3.83, respectively. And the Fold Change of aly-miR166g-5p, ath-miR167d, osa-miR166d-5p, and ath-miR393a were less than −4. In total, 21.3 % potential targets were regulated through translational repression but 78.7 % were cleavage. Twenty-five unique miRNAs and their 20 target mRNAs presented a positive correlation, while 41 miRNAs and their 30 target mRNAs presented an anti- correlation. Among them, ptc-miR399j only presented in dwarf mutant (A1), and its target gene comp27522_c0_seq1 (protein-serine/threonine kinase) changed by −1.41 fold. Furthermore, ath-miR858b changed by −1.2 fold and its target gene comp285359_c0_seq1 (myb proto-oncogene protein) changed by 2.5 fold (Additional file 5). In dwarf mutant (A1) compared with its wild type (A9), miR166 family, including cme-miR166i, gma-miR166h-3p, gma-miR166m, gma-miR166u, osa-miR166e-3p, sbi-miR166k, and zma-miR166h-3p, was down-regulated and targeted 21 genes, including 7 homeobox-leucine zipper protein (comp45076_c0_seq1), 4 peroxidase (comp44152_c0_seq7), 4 ABA responsive element binding factor (comp138319_c0_seq1 and comp30734_c0_seq3), 3 ribonucleoside-diphosphate reductase subunit M2 (comp35879_c0_seq3) (Additional file 6).

Between tall-culm (A3) and its wild type (A9) (Additional file 7), 74 miRNAs with 166 annotated targets differentially expressed. Among them, 10 miRNAs were up-regulated but 64 miRNAs were down-regulated. The fold Change of aly-miR396a-3p, pab-miR3711, ath-miR396b, and ath-miR171b was more than 4 with 4.43, 4.49, 4.95 and 5.01, respectively. And only the fold Change of ath-miR408 was less than −2. In total, 21.5 % potential targets were regulated through translational repression but 78.4 % were cleavage. Thirty-two unique miRNAs and their 17 target mRNAs presented a positive correlation, while 13 miRNAs and their 11 target mRNAs presented an anti-correlation. Among them, ath-miR159a changed by −1.81 fold, and its target gene comp189595_c0_seq1 (cytokinin dehydrogenase) changed by −1.16 fold; ptc-miR396g-5p changed by 2.25 fold, and its target gene comp33348_c0_seq1 (IAA-amino acid hydrolase) changed by 1.82 fold. Furthermore, gma-miR166u changed by 1.4 fold and its target gene comp138319_c0_seq1 (ABA responsive element binding factor) changed by −1.86 fold; osa-miR159f changed by −1.70 fold and its target gene comp215064_c0_seq1 (pectatelyase) changed by 1.83 fold. In A3-A9, miR166 family, including ath-miR166a, bdi-miR166e, cme-miR166i, crt-miR166b, crt-miR166, gma-miR166h-3p, gma-miR166m, gma-miR166u, mtr-miR166b, osa-miR166e-3p, osa-miR166g-3p, osa-miR166m, sbi-miR166k, and zma-miR166h-3p, was down-regulated and targeted 42 genes, including 14 homeobox-leucine zipper protein (comp45076_c0_seq1), 10 peroxidase (comp44152_c0_seq7), 4 ABA responsive element binding factor(comp138319_c0_seq1 and comp30734_c0_seq3), 7 ribonucleoside-diphosphate reductase subunit M2 (comp35879_c0_seq3). And ABA responsive element binding factor (comp138319_c0_seq1) was up-regulated (Additional file 6).

Only 24 miRNAs and 13 miRNAs were only detected in dwarf mutant (A1), and tall-culm mutant (A3), respectively. Of these miRNAs, miR171 were the most abundant miRNA family both expressed in dwarf mutant (A1) and tall-culm mutant (A3). Targets of all the 37 specific expressed miRNAs were identified. In dwarf mutant (A1), four AP2-like factors, one transcriptional regulator ATRX and one cytochrome P450 were targeted by miR172, one U4/U6 small nuclear ribonucleoprotein SNU13 and one tubulin beta were targeted by miR171, and two myb proto-oncogene protein, one CDK-activating kinase assembly factor MAT1 and glutathione S-transferase were targeted by miR828 (Additional file 8). The targets of miRNAs only expressed in tall-culm mutant (A3) include two myb proto-oncogene proteins and one translation initiation factor 2 targeted by miR159. Unfortunately, functions of the targets of some miRNAs are currently unknown.

Besides, the targets for predicted novel miRNAs were also identified. Total 36 annotated targets for 38 novel miRNAs were identified. Functions of the target genes were various, such as extracellular signal-regulated kinase 1/2 targeted by ghr-m0444-3p, auxin efflux carrier family targeted by ghr-m3978-5p, EREBP-like factor targeted by ghr-m3978-5p, protein smg7 targeted byghr-m1352-3p, ghr-m3726-3p, ghr-m3818-3p, ghr-m4247-3p, ghr-m4971-3p and ghr-m3014-3p, and protein phosphatase 2C targeted by ghr-m1071-3p (Additional file 9).

### Location and annotation of target genes of miRNAs in cotton AD genome

Fifty-one miRNAs and their targets related to dwarf mutant and 34 miRNAs related to tall-culm mutant were obtained, respectively. Fifty-one target genes related to dwarf mutant were involved in 18 chromosomes and 5 scaffolds. Twenty target genes located on the A_t_ chromosomes and the 25 target genes located on the D_t_ chromosome were related to dwarf mutant (Additional file 10). The value of log_2_ (A1-A9) of target genes comp439597_c0_seq1, comp202465_c0_seq1, and comp1410_c0_seq1 were more than 8, while that of comp400392_c0_seq1, comp419770_c0_seq1, and comp444052_c0_seq1 were less than −8 in A1-A9. Thirty-two target genes related to tall-culm mutant were involved on 14 chromosomes. Fourteen target genes located on the A_t_ chromosomes and the 15 target genes located on the D_t_ chromosome were related to dwarf mutant (Additional file 10). The value of log_2_ (A3–A9) of target genes comp266331_c0_seq1, comp439597_c0_seq1 and comp202465_c0_seq1 were more than 8, while comp231082_c0_seq1 and comp202109_c0_seq1 were less than −8 in A3–A9. Genomic signatures of selection and domestication are associated with positively selected genes for fiber improvement on the A subgenome and for stress tolerance on the D subgenome [27, 28]. In this study, we found that there were more genes related to dwarf mutant in the D_t_ subgenome.

### Expression pattern analysis of miRNAs and their targets

**Fig. 6:**
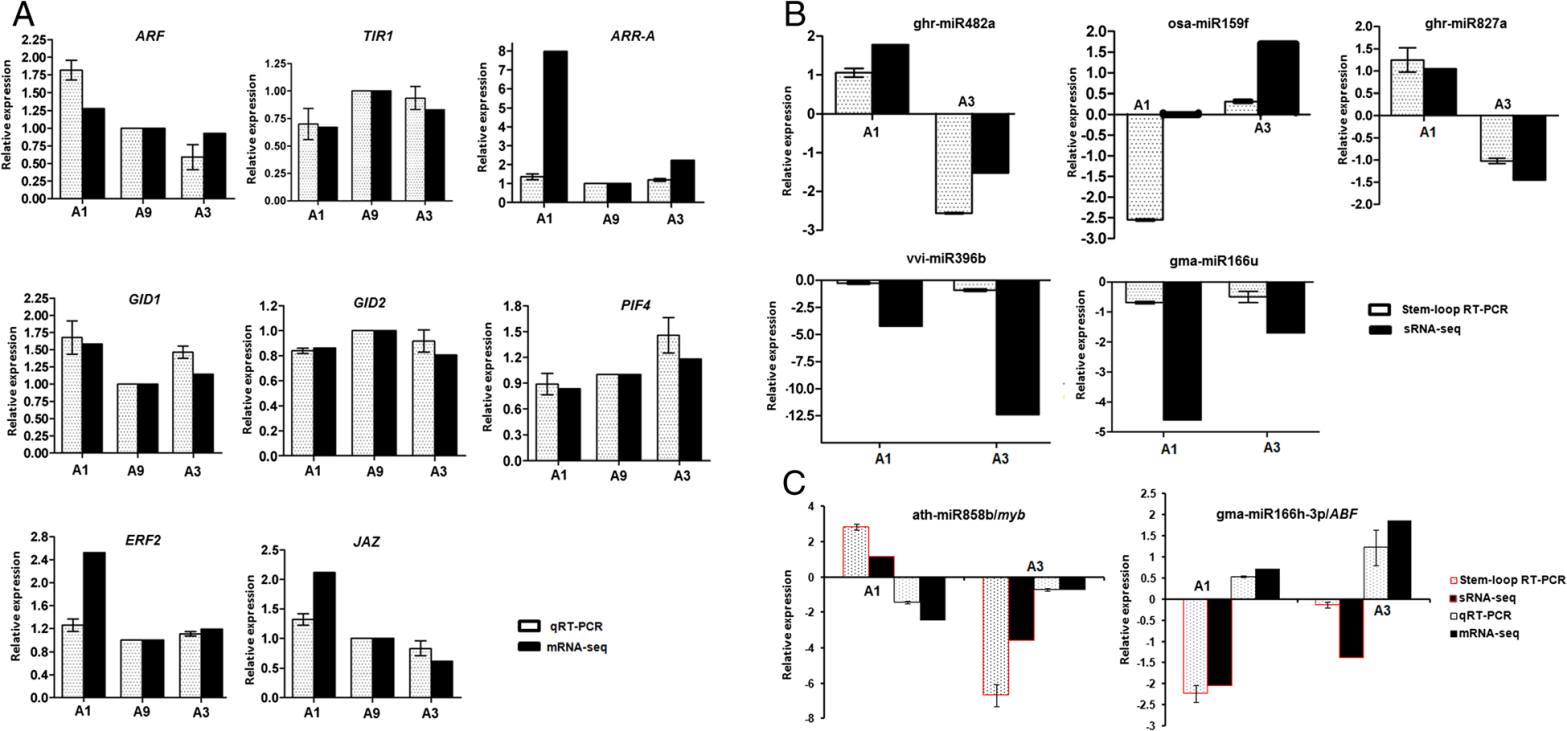
Validation of the expression patterns of the predicted DEGs and miRNAs by qRT-PCR and stem-loop qRT-PCR in dwarf mutant and tall-culm mutant. **a** 8 DEGs were validated by qRT-PCR in the mutants compared with that of wild type. **b** The expression patterns of five miRNAs were confirmed using stem-loop qRT-PCR. **c** Two miRNAs and their targets were validated using qRT-PCR and stem-loop qRT-PCR. The white bars represent the relative expression levels of stem-loop qRT-PCR or qRT-PCR. The equation ratio = 2^△△Ct^ was applied to calculate the expression level relative to wild type Ari971, using *GhUBQ7* as the reference gene. Three biological replicates and three technical replicates were performed. The black bars represent [Log_2_RPM A1 (A3)–A9] of data of sRNA-seq. A1: Ari1327, dwarf mutant; A3: Ari3697, tall-culm mutant, A9: wild type Ari971

To confirm the unigenes obtained from sequencing, eight unigenes related to hormone were chosen for qRT-PCR analysis. These qRT-PCR results were consistent with those obtained from the DEG expression profiling. For example, the expression of *GID1* was both up-regulated in A1 and A3 as compared with A9. However, *GID2* showed an opposite tendency (Fig. 6a). Then, the expression levels of randomly selected representative miRNA species were further determined through stem-loop qRT-PCR. Results showed that the relative abundance of almost all the miRNAs determined by stem-loop qRT-PCR followed similar trends as the read numbers in the libraries. As shown in Fig. 6b, three miRNAs (vvi-miR396b, gma-miR166u, and gma-miR166h-3) were down regulated both in A1–A9 and A3–A9, three miRNAs (ghr-miR482a, ath-miR858b, and ghr-miR827a) up-regulated in A1–A9 but down-regulated in A3–A9, and one miRNA (osa-miR159f) down regulated in A1–A9 but up-regulated in A3–A9. In addition, two miRNA and their targets were validated using qRT-PCR and stem-loop qRT-PCR. As shown in Fig. 6c, ath-miR858b was up-regulated in A1–A9 but down-regulated in A3–A9; its target *myb* gene (comp285359_c0_seq1) was down-regulated in A1–A9, but it did not change significantly in A3–A9. The gma-miR166h-3p was down-regulated while its target *ABF* was up-regulated both in A1 and A3. Both the two results of the qRT-PCR and RNA sequencing were consistent.

### Validating miRNA/target interactions using PAREsnip

PAREsnip program was utilized to validate the different expressed miRNA/target interactions and make an in-depth survey of cleavage sites in transcripts and corresponding small RNAs. In the dwarf mutant (set 1,A1–A9), PAREsnip reported 42 interactions. The 42 interactions are shown in Additional file 11. When comparing the results of sets 1 and our sequenced miRNA/target, we validated a total of 27 conserved interactions between small RNAs and corresponding transcript genes. There were 4 miR172/AP2 factor, 4 miR160/comp38451_c0_seq2 (auxin mediated signaling pathway), 3 miR159/ myb proto-oncogene protein, 2 miR319/myb proto-oncogene protein, 1 miR858/myb proto-oncogene protein, and 1 ath-miR393a/ comp45893_c0_seq5 (auxin mediated signaling pathway) (Additional file 12) . In the tall-culm mutant (set 2,A3–A9), PAREsnip reported 72 interactions. These interactions are shown in Additional file 11. When comparing the results of sets 2 and our sequenced miRNA/target, 29 conserved interactions were validated. There were 2 miR172/AP2 factor, 4 miR159/myb proto-oncogene protein, 3 miR160/comp38451_c0_seq2 (auxin mediated signaling pathway) and 1 miR858/ myb proto-oncogene protein (Additional file 12). Comparing the two sets, we found that miRNA160 and miRNA172 were up-regulated in A1–A9 but down-regulated in A3–A9, whereas miRNA159 showed the opposite regulation. In addition, 2 miR319/ myb proto-oncogene protein were up-regulated and only expressed in A1–A9. These data suggest that miRNA160, miRNA172, miRNA159, miR319 etc. maybe regulate the plant height mutation in cotton.

## Discussion

Previous studies of dwarf mutants indicate that plant hormones, such as GA [29] and brassinosteroids (BR) [30], play significant roles in plant height decision. Thus, the mutants in plant hormones synthesis or signaling show dwarf phenotypes: The semi-dwarf rice results from *sd1* gene, which encodes a GA20oxidase (*OsGA20ox2*), and *dwarf18*(*d18*) dwarves result from mutations in a GA3ox gene (*OsGA3ox2*); Loss of function mutations in *GID1* or *GID2* result in dwarf phenotypes, whereas *GID1* overexpression causes GA hypersensitivity and internodes elongation. Reduction in expression of *ZmDWF1*, a maize homolog of *DIM1/DWF1*, resulted in dwarf maize plants [31]. Furthermore, plant height variation was controlled by quantitative trait loci (QTL): loci *Rht-D1* and *Rht-B1* had the largest effects on plant height in European winter wheat cultivars and that other small- or medium-effect QTL (*Ppd-D1* and *Rht8*) and potentially epistatic QTL enable fine adjustments in plant height [32]; Many QTLs, including *QPh.cgb-2D.1*, *QPh.cgb-4B.1*, *QPh.cgb-4D.1*, and *QPh.cgb-5A.7* coincident with reduced height (*Rht*) genes (*Rht8*, *Rht1*, *Rht2*, and *Rht9*), interacted with more than one other QTL, indicating that the genetic architecture underlying plant height development is a network of genes with additive and epistatic effects [33]. In our previous study, two QTLs *qPH-LG6-1* and *qPH-11-1* for the plant height of the drawf mutant Ari1327 were found with a distance of 2.01 and 0.02 cM to the nearest marker of NAU4073 and DPL0570 respectively [34]. In this study, miRNA and their target genes related to plant hormones (IAA and ABA), cell growth and redox reactions were involved in the cotton height.

### IAA and ABA was related to the plant height in cotton

Apical dominance in a primary shoot or inflorescence inhibits axillary meristem growth. Measurement and exogenous application of plant hormones and the generation of mutants with altered hormone levels have determined both the inhibitory and promoting effects of auxin in controlling apical dominance [35]. The phytohormone auxin regulates plant growth and development by controlling the fundamental processes of cell division, expansion, and differentiation. Additional studies have demonstrated that IAA is important during plant growth and development. However, while IAA in low concentrations stimulates growth and development, higher concentrations can be toxic to the plant [36]. Therefore, tight control of IAA concentration is necessary for proper plant development. The IAA content of dwarf mutant (A1) was higher than that of the wild type (A9) in our study.

The transcriptome analyses showed that plant hormone signal transduction pathway was enriched of A1–A9 and A3–A9 with *p*-value as 8.6E-21 and 2.08E-14, respectively. Auxin exerts its regulatory role, at least to an extent, by rapidly inducing a group of genes that are collectively termed as early auxin response genes. These genes are categorized into three major classes: *Aux*/*IAA*s, *SAURs* and *GH3s* [37].

*SAUR* genes, which currently count 82 members together with *SAUR*-like genes on the TAIR web site (https://www.arabidopsis.org/), encode small proteins with estimated molecular masses of 9–12 kDa. Although the biochemical or developmental functions of this family remains largely unknown, the members have been reported to accumulate within 2.5 min after auxin treatment [38], to be correlated with elongating tissues [39, 40] and to negatively influence synthesis of auxin and proteins for polar auxin transport [41, 42]. Recent studies of the *SAUR19* and *SAUR63* subfamilies have implicated these *SAURs* as positive effectors of cell expansion [39, 40, 43]. SAUR19 family proteins increased hypocotyl and leaf size, altered tropic responses, and defects in apical hook maintenance [39]. Likewise, SAUR63 fusion proteins confer several cell expansion phenotypes including increases in hypocotyl, petal, and stamen length [40]. In the present study, *SAUR* was down-regulated with the fold change as −3.37 in A1–A9, while the fold change was 2.48 in A3–A9. It was suggested that lower expressed *SAUR* inhibited the plant growth in dwarf mutant.

The *GH3* genes encode a group of enzymes that adenylates IAA, salicylic acid, or jasmonic acid [44]. The GH3 enzymes also conjugate free IAA with amino acids. Consistent with the biochemical activities, *Arabidopsis* mutants with elevated *GH3* expression, such as *dfl1-D* [45], and *dfl2-D* [46], display reduced growth and altered leaf shape. In dwarf mutant, the fold change of *GH3* showed 3.15,while it was −2.67 in high mutant, compared with wild type. It was referred that high abundant *GH3* inhibited the plant growth in cotton.

The auxin signaling pathway requires the auxin response factor (*ARF*), such as *ARF10*, *ARF16*, and *ARF17* in *Arabidopsis* which are the targets of miRNA160. Plants expressing miR160-resistant *ARF17* exhibited pleiotropic developmental defects, including abnormal stamen structure and reduced fertility [47]. In this paper, miRNA160 were validated to be up-regulated in dwarf-mutant but down-regulated in tall-mutant. It suggested that miRNA160 played a negative regulation role in regulating cotton height.

Taken together, these results suggest lower expressed *SAUR* and elevated expressed *GH3* maybe relate to reduced growth of the plant height in the dwarf mutant (A1) which is consistent with the higher auxin content in this mutant. Furthermore, miRNA160 target to *ARF* in a reverse regulation way in the dwarf and tall-culm mutant indicated the mutation related to the auxin signaling pathway. However, how auxin regulated the cotton plant height through *SAUR* and *GH3* regulated by the miRNA160 need further researches.

In the carotenoid biosynthesis pathway, both in the dwarf mutant (A1) and the tall-culm mutant (A3) compared with the wild type (A9), ABA related genes had the greatest change. *NCED* was down-regulated with fold change as −4.2 in A1–A9, but up-regulated with 3.5 fold in A3–A9. *ABA2* and *AAO3* were both down-regulated in A3–A9, but they were not different significantly in A1–A9. It has been documented that a rate-limiting step in ABA biosynthesis is the oxidative cleavage of 9-*cis*-epoxycarotenoid to produce xanthoxin, catalyzed by NCED in plastids. In the cytosol, xanthoxin dehydrogenase catalyzes the conversion of xanthoxin to abscisyl aldehyde, which is converted to ABA by aldehyde oxidase [48]. Recent key studies in the vascular plant *Arabidopsis* provide evidence of a pivotal role of PP2Cs in the ABA signaling pathway. In the absence of ABA, Group A PP2Cs act through the physical interaction with subclass III of plant-specific SnRK2 and dephosphorylate the kinase activation loop, inhibiting ABA signaling transduction. It is evident that Group A PP2C is a central component in the induction of ABA signaling and a negative regulator of abscisic acid signaling [49]. In A1–A9, *PP2C* was up-regulated with fold change as 4.6, while it was down-regulated with fold change as −2.6 in A3–A9. Furthermore, an up-regulated ABA responsive element binding factor (comp138319_c0_seq1) was targeted by gma-miR166u and gma-miR166h-3p down-regulated in the tall-culm mutant (A3) compared with the wild type (A9).

Another ABA responsive element binding factor (comp30734_c0_seq3) was also targeted by gma-miR166u and gma-miR166h-3p. MiR166 and its targets regulate an array of plant developmental processes, including shoot apical and lateral meristem formation, leaf polarity, floral development, and vascular development. Two activation-tagged mutants overproducing miR166 have recently been characterized. The men1 mutant overexpressing the miR166a gene exhibited pleiotropic phenotypes, such as stunted growth, disrupted floral structure, fasciated inflorescence stem, and enlarged shoot apical meristem (SAM) [50]. The jba-1D mutant, in which the miR166g gene is activation-tagged, has also been isolated from an activation-tagging approach [51]. The mutant exhibited essentially identical phenotypes to those observed in the men1 mutant. It was demonstrated that miR166 plays a role in regulating meristem formation. In this work, both in A1–A9 and A3–A9, the miR166 family were down-regulated. However, all the targeted genes did not change significantly except comp138319_c0_seq1 (ABA responsive element binding factor) in A3–A9. It was referred that gma-miR166u and gma-miR166h-3p, the members of miR166 family, and their targeted gene, ABA responsive element binding factor, were related to the plant height mutation.

### Cell growth related factors was involved in the plant height of cotton

*smg7*, which are implicated in nonsense-mediated RNA decay and in telomere metabolism, is crucial for completion of the meiotic cell cycle. Two T-DNA insertion mutants of *smg7*: *smg7-1* and *smg7-3* exhibited severe growth retardation [52]. In this work, the expression of *smg7* did not change significantly in A3–A9. However, it was down-regulated in A1–A9. Conserved miRNA gra-miR482, which targeted *smg7*, was up-regulated in A1–A9 while it was down-regulated in A3–A9. Total 6 of 38 novel miRNAs, including ghr-m1352-3p, ghr-m3014-3p, ghr-m3726-3p, ghr-m3818-3p, ghr-m4247-3p, ghr-m4971-3p, targeted *smg7*. All of them were up-regulated in A1–A9, but did not change significantly in A3–A9. It was referred that gra-miR482, the novel miRNAs and their targeted gene *smg7* were related to dwarf mutant.

Pectate-lyases have previously been described as microbial extracellular enzymes that assist pathogenesis by cleaving of polygalacturonate blocks in the plant host cell wall. However, *in situ* hybridization studies in young *Zinnia* stems show that *pectate-lyases* expression was associated with vascular bundles and shoot primordia. The mRNA encoding this enzyme is up-regulated *in vitro* during both cell elongation and cell differentiation in response to auxin [53]. In this work, *pectate-lyase* was down-regulated in A3–A9. However, it did not change significantly in A1–A9. And *pectate-lyase* was targeted by osa-miR159f, which was up-regulated in A3–A9. MiR159 family including aqc-miR159, ath-miR159a, osa-miR159f, pde-miR159, pta-miR159a, ath-miR159c, and pta-miR159c, are related to axillary bud outgrowth [54], salt and drought stresses [55], flowering time [56], and ethylene treatment [57]. According to our results, miR159 may also regulate the plant height.

### Redox reactions related factors was involved in the plant height of cotton

Cytochrome P450 (comp39936_c0_seq2), a family of membrane-bound heme-containg proteins in both eukaryotic and prokaryotic organisms, mediated a wide range of redox reactions involved in the biosynthesis of plant hormones and secondary metabolites including ABA [58], GA, BR, lignins, UV protectants, pigments, defense compounds, fatty acids and signaling molecules [59]. In our study, *Cytochrome P450* was targeted by gma-miR172k and osa-miR172c, which were both up-regulated in the dwarf mutant (A1), but could not be detected in the tall-culm mutant (A3) compared with the wild type (A9). It was inferred that miR172 might be involved in dwarf mutant through targeting *cytochrome P450* to interfere with the biosynthesis of terpenoids, such as GA, ABA, carotenoids and defensive substance of plant [60]. Wang et al. [61] discovered that *cytochrome P450* was targeted by cotton-specific miRNA, miR2948-5p, indicating that the *Cytochrome P450* families were targeted by different miRNAs in cotton.

### *MYB* gene was involved in the plant height of cotton

The *MYB* gene family is one of the largest families in plant kingdom, and some of its members are regulated by miRNAs. Major functions of *MYB* in *Arabidopsis* include primary and secondary metabolism, cell fate and identity, developmental processes and responses to biotic and abiotic stresses [62]. It has been demonstrated that *MYBs* were targeted by miR159, miR828 and miR858 in *Arabidopsis* [63] and apple [64]. The miR828 targeted two *MYB* genes, and one of which was *GAMYB,* a *MYB* transcription factor involved in GA signal transduction [65, 66]. It induced downstream genes expression through combining with promoters of GA-responsive gene. In this study, two *MYB* related genes was found to be targeted by miR828 in dwarf mutant (A1). Moreover, the down-regulated myb proto-oncogene protein was targeted by up-regulated ath-miR858b in A1–A9, but it did not change significantly in A3–A9. Another two *MYB* genes targeted by miR159 in tall-culm mutant (A3), which maybe involved in a negative pathway to control plant height. Therefore, it was suggested that both miR828 and miR858 targeting the *MYB* genes in dwarf mutant (A1) may contribute to the dwarfism of cotton plant. In addition, it was found recently that miR828 and miR858 regulate homoeologous *MYB2* gene functions in cotton fiber development [67]. *MYB2* gene promotes cotton fiber development and is functionally homologous to *Arabidopsis GLABROUS1* (*GL1*) in trichome formation. Therefore, miR828 and miR858 not only regulated for plant height development, but also related to fiber development.

## Conclusion

Plant height is an important trait in cotton. In this study, we try to reveal the networks involved in regulation mechanism of plant height in *Gossypium hirsutum* by comparision analysis of transcriptome and small RNA sequencing of stem apexes at the fifth true leaf stage among the dwarf-mutant (A1), tall-mutant (A3) and their wild type (A9). The transcriptome sequencing results showed that carotenoid biosynthesis, plant-pathogen interaction and plant hormone signal transduction were the top 3 DEGs enriched pathways in both dwarf mutant (A1) and the tall-culm mutant (A3). The ABA and IAA related factors were differentially expressed in the mutants. In the pathway, the ABA related factors of *ABA2* and *AAO3* were the top two down-regulated genes in the tall-culm mutant compared with the wild type (A3–A9), but they did not change significantly in dwarf mutant (A1–A9). However, *SAUR* and *GH3* which were related to IAA regulation showed the change by a contrary tendency. Of the 226 known conserved miRNAs, 20 were identified as known cotton miRNAs. A total of 104 miRNAs in A1–A9 and 100 miRNAs in A3–A9 were identified to be differentially expressed. Moreover, 97 miRNAs with 153 annotated targets different expressed between A1 and A9, and 74 miRNAs with 166 annotated targets differentially expressed between A3 and A9. Furthermore, we have noticed that miRNA166, targeting ABA related factors, were both down- regulated in A1–A9 and A3–A9; miR172 might be involved in dwarf mutant through targeting *cytochrome P450*; miR828, miR858 and miR159 targeting the *MYB* genes may contribute to plant height. Using PAREsnip, miRNA160/auxin related factors, miRNA172/AP2 factor, miR858/myb proto-oncogene protein, and miR159/myb proto-oncogene protein were validated. In addition, miRNA160, miRNA858 and miRNA172 were validated to be up-regulated in A1–A9 but down-regulated in A3–A9, whereas miRNA159 showed the opposite regulation. This work laid a foundation to elucidate further function of miRNAs and how they interact with their targets in regulating the plant height.

## Methods

### Plant materials and growth conditions

Ari1327 was a dwarf mutant and Ari3697 was a higher plant mutant by ^60^Co γ-ray irradiation from an American upland cotton line, Ari971. The seeds of upland cotton, Ari971, wild type (*G. hirsutum* cv.) and its dwarf mutant (Ari1327) and tall-culm mutant (Ari3697) were available in the National Mid-term Genebank of the Institute of Cotton Research, Chinese Academy of Agricultural Sciences and surface sterilized in 30 % H_2_O_2_ for 3 h, washed with distilled water for three times, and then soaked in distilled water for 1 day at room temperature. Sterilized seeds were grown and maintained in pots in a greenhouse of Institute of Cotton Research, Chinese Academy of Agricultural Sciences at a day/night temperature of 28/22 °C with a 14-h photoperiod. At the fifth true leaf stage, stem apexes of wild type and mutant seedlings were harvested and immediately frozen in liquid nitrogen and stored at −80 °C until further use. The plant height and internodes length were measured use a ruler and recorded before collected the apexes.

### Construction of small RNA and cDNA libraries

Total RNA was purified from stem apexes of three samples (Ari971, Ari1327 and Ari3697) using Trizol reagent (Invitrogen) according to the manufacturer’s protocols. Equal amounts of RNA from Ari971, Ari1327 and Ari3697 were pooled for transcriptome and small RNA libraries construction. The transcriptome library was prepared using an Illumina TruSeq RNA Sample PreKit following the manufacturer’s instructions. After removing adaptors and low-quality reads, mRNA transcriptome *de novo* assembly was performed using the Trinity (trinityrnaseq_r2012-10-05, https://en.osdn.jp/projects/sfnet_trinityrnaseq/releases/) program. Three small RNA libraries from Ari971, Ari1327 and Ari3697 were prepared based on a previously described procedure [68]. Briefly, sRNA fragments ranging from 17 to 35 nucleotides (nt) were separated and purified by polyacrylamide gel electrophoresis and ligated to 5′- and 3′-RNA adaptors by T4 RNA ligase (TaKaRa). The adaptor-ligated sRNAs were subsequently transcribed to single-stranded cDNA using SuperScript II Reverse Transcriptase (Invitrogen). Both small RNA and transcriptome sequencing were performed on Illumina Genome analyzer (Sangon Biotech, Shanghai, China).

### Analysis of small RNA sequencing data

Clean reads were screened from raw sequencing reads by removing contaminated reads including sequences with 5′-primer contaminants, without the inserted tag, with poly (A) tails, either shorter than 17 nt or longer than 35 nt. The clean sequences matched to non-coding tRNAs, rRNAs, small nuclear RNA (snRNAs) and small nucleolar RNA (snoRNAs) with a maximum of one mismatch mapped to the tRNAdb, SILVA rRNA and NONCODE v3.0 database were removed. The remaining unique sequences were aligned with known miRNAs from miRBase 19.0 (http://www.mirbase.org/index.shtml) with no mismatch to identify the known/conserved miRNAs. The remaining unknown reads were used for prediction of novel miRNAs by align against genome sequences of *G. raimondii*.

### Identification of conserved miRNAs and prediction of novel miRNAs in cotton

Sequences in the small RNA libraries with no mismatch and more than 16 matches to currently known miRNAs from all plant species were regarded as potential reference miRNAs of a known miRNA family. Precursors of known miRNAs and novel miRNA candidates were identified by extracting 150 nt of the sequence flanking the contig sequences matching the known miRNAs and unannotated small RNAs by analyzing their secondary structural features using the MIREAP pipeline (https://source-forge.net/projects/mireap/). Basic criteria [69] were used for screening the potential novel miRNAs. RNAs with the characteristic hairpin structure, with minimal matched nucleotide pairs of miRNA and miRNA* exceeding 16 nt and with maximal size differences of miRNA and miRNA* up to 4 nt, were retained as precursor of known miRNAs or novel miRNAs. Novel miRNAs were predicted by MIREAP, and the minimal folding free energy index (MFEI) of precursors greater than 0.85 were then predicted by Mipred and triplet-svm-classifier. The miRNA precursor matched above two conditions were identified as a real one. The stem-loop structures of pre-miRNAs were constructed by MFOLD and selected manually [70].

### Differential expression analyses of miRNAs between wild type and mutant

The frequency of miRNAs from three libraries was normalized as ‘reads per million’ (RPM = mapped reads/total reads × 1,000,000). If the normalized read count of a given miRNA is zero, the expression value was modified to 0.001 for further analysis. The fold change between the wild type and mutant library was calculated as: fold change = log_2_ (A9–A1 or A3). The miRNAs with fold changes > 2 or <0.5 and with *p* ≤ 0.05 were considered to be up-regulated or down-regulated in response to mutant, respectively. The *p*-value was calculated according to previously established methods [71, 72]. The locations of differentially expressed target genes of these miRNAs were analyzed using blastn with default values.

### Real-time qPCR

The mature miRNA reverse transcription was performed with miR-specific stem-loop forward primers and a universal reverse primer, URP. These miR-specific primers were designed according to the mature miRNA sequence [73] (Additional file 13). For real-time PCR, cDNA was mixed with 2 × SYBR Green Mix (Takara, Japan) and each of the miRNA specific primers and universal reverse primer in a final volume of 20 μl. The PCR process was performed using SYBR Green as fluorescence dye and run on 96-wells plates with the ABI 7500 FAST Real Time system (Applied Biosystems). PCR was performed with 95 °C for 30 s, 40 cycles of 95 °C for 3 s, 60 °C for 30 s and 72 °C for 30 s. *Ubiquitin 7* (*GhUBQ7*) (Additional file 13) was used as the reference gene [74]. The relative expression level of miRNA was calculated by 2^-△△Ct^. Three biological replicates were performed.

### Validating miRNA/target interactions using PAREsnip

To validate the miRNA/target interactions, PAREsnip program was used to analyze the following data sets: different expressed miRNAome (A1 versus A9), degradome (GSM1061853), transcripts (A1 and A9) (set 1); different expressed miRNAome (A3 versus A9), degradome (GSM1061853), transcripts (A3 and A9) (set 2). For every subsequent analysis, the following settings were used: a maximum of 4.0 mismatches, 100 dinucleotide shuffles and a *P*-value threshold of 0.05.

### Availability of supporting data

The data sets supporting the results of this article are available in the GEO repository (accession ID: GSE71608).

## Additional files

Additional file 1:
**The classification of the large-scale short reads into known categories.** (TIFF 83 kb) Additional file 2:
**Distribution of miRNAs in different families.** (TIFF 1216 kb) Additional file 3:
**Twenty nine precursors identified using miRDeep 2.** (PDF 1319 kb) Additional file 4:
**Novel miRNA identified in all libraries.** (XLSX 12 kb) Additional file 5:
**Predicted targets of miRNAs different expressed between dwarf mutant (A1) and wild-type (A9).** (XLSX 86 kb) Additional file 6:
**miR166 family and the targets in dwarf mutant (A1) and tall-mutant (A3).** (XLSX 16 kb) Additional file 7:
**Predicted targets of miRNAs different expressed between tall-mutant (A3) and wild-type (A9).** (XLSX 82 kb) Additional file 8:
**miRNAs and their targets only identified dwarf mutant (A1) or tall-mutant (A3).** (XLSX 31 kb) Additional file 9:
**Annotated targets of novel miRNAs.** (XLSX 34 kb) Additional file 10:
**The location of miRNA target genes related to dwarf mutant and tall-culm mutant in cotton AD genome.** (XLSX 326 kb) Additional file 11:
**The miRNA/target interactions validated by PAREsnip program.** (XLSX 17 kb) Additional file 12:
**The Illustration of small RNAs and target transcripts.** Scatter plot diagrams show the frequency of tags and their positions on transcripts. The inferred cleavage sites were indicated by the Blocks and dotted line. (PDF 18 kb) Additional file 13:
**Stem-loop qRT-PCR primer characteristics.** (XLSX 10 kb)

## Abbreviations

A1: Ari1327
A3: Ari3697
A9: Ari971
AAO3: Abscisic-aldehyde oxidase
ABA2: Xanthoxin dehydrogenase
ARF: Auxin response factor
BL: Brassinolide
BR: Brassinosteroids
DEG: Differentially expressed gene
GA: Gibberellins
GO: Gene ontology
KEGG: Kyoto encyclopedia of genes and genomes
QTL: Quantitative trait loci
RPM: Reads per million
SAM: Shoot apical meristem
