# Supplementary figures and images for: MicroRNA and mRNA expression profiling analysis revealed the regulation of plant height in Gossypium hirsutum
Source: BMC Genomics. 2015 Oct 30;16:886. doi: 10.1186/s12864-015-2071-6 (PMC4628322; doi:10.1186/s12864-015-2071-6)

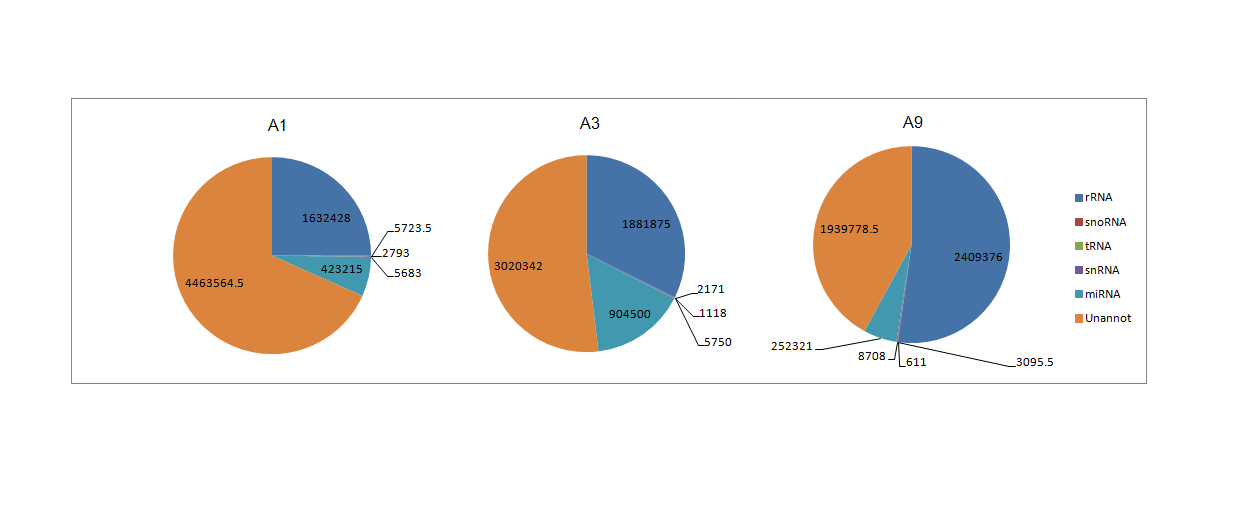

Supplement: Additional file 1: — The classification of the large-scale short reads into known categories. (TIFF 83 kb) [file 12864_2015_2071_MOESM1_ESM.tif]

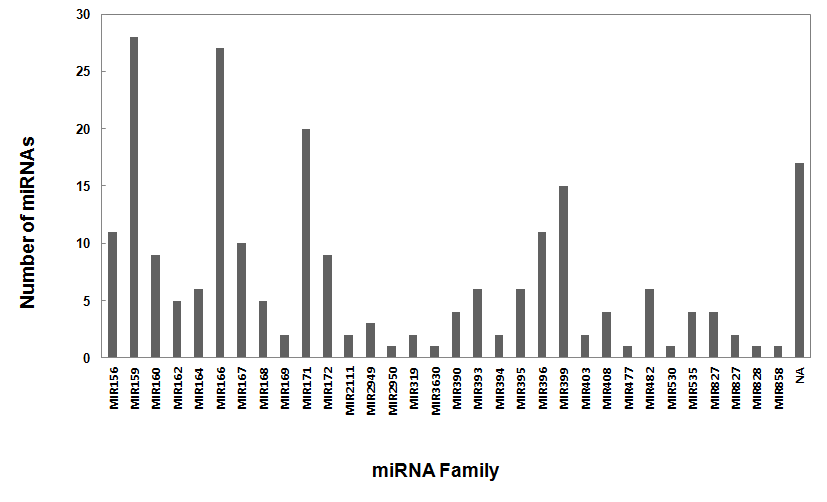

Supplement: Additional file 2: — Distribution of miRNAs in different families. (TIFF 1216 kb) [file 12864_2015_2071_MOESM2_ESM.tif]
