# Supplementary material for: MicroRNA and mRNA expression profiling analysis revealed the regulation of plant height in Gossypium hirsutum
Source: BMC Genomics. 2015 Oct 30;16:886. doi: 10.1186/s12864-015-2071-6 (PMC4628322; doi:10.1186/s12864-015-2071-6)

miR164f miR164g-3p miR167i miR169n miR171b-1 miR171b-2 miR171b-3 miR171b-4

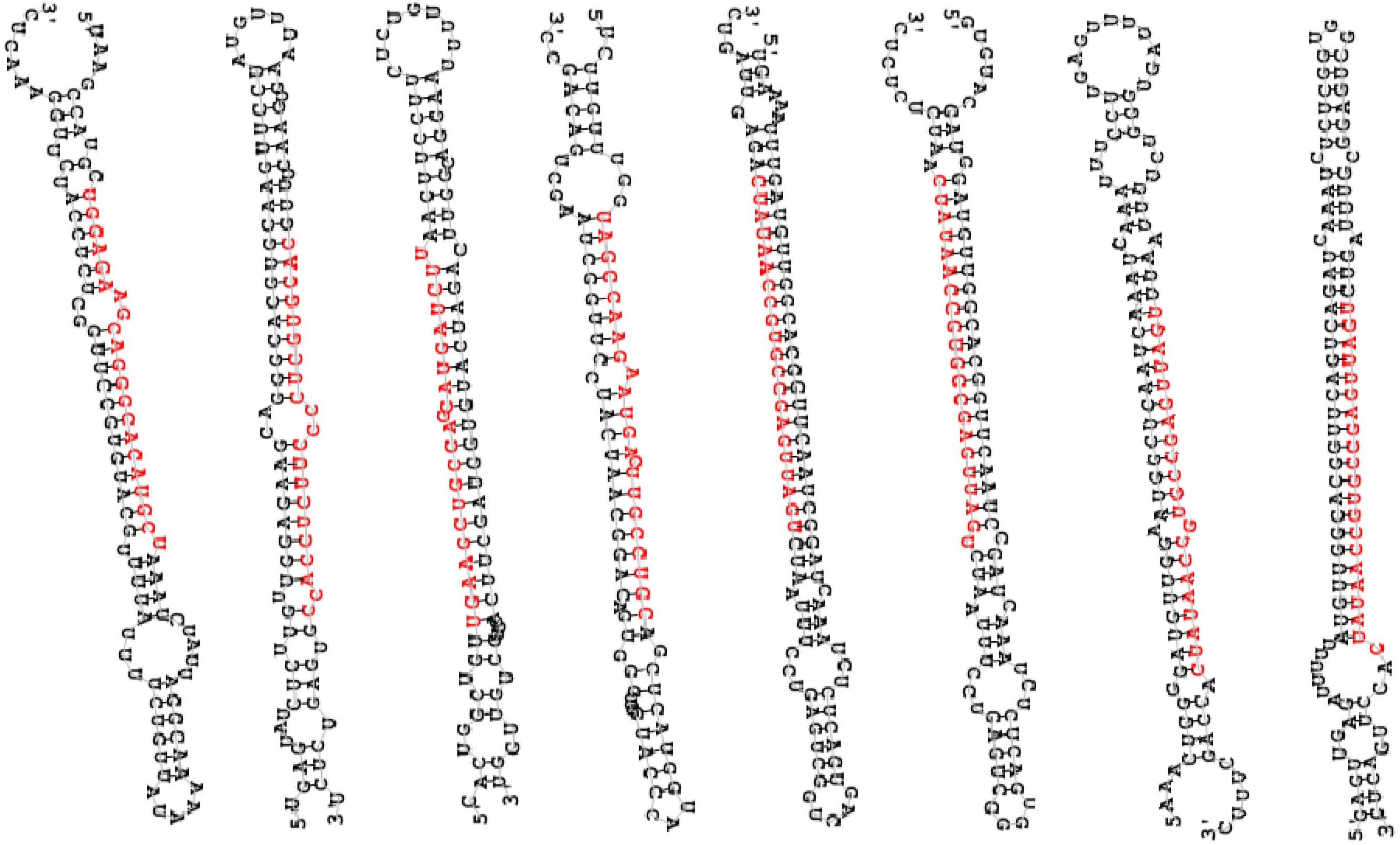

miR171b-5 miR390a miR393b-3p miR395a miR399a-1 miR399a-2 miR399b miR399d

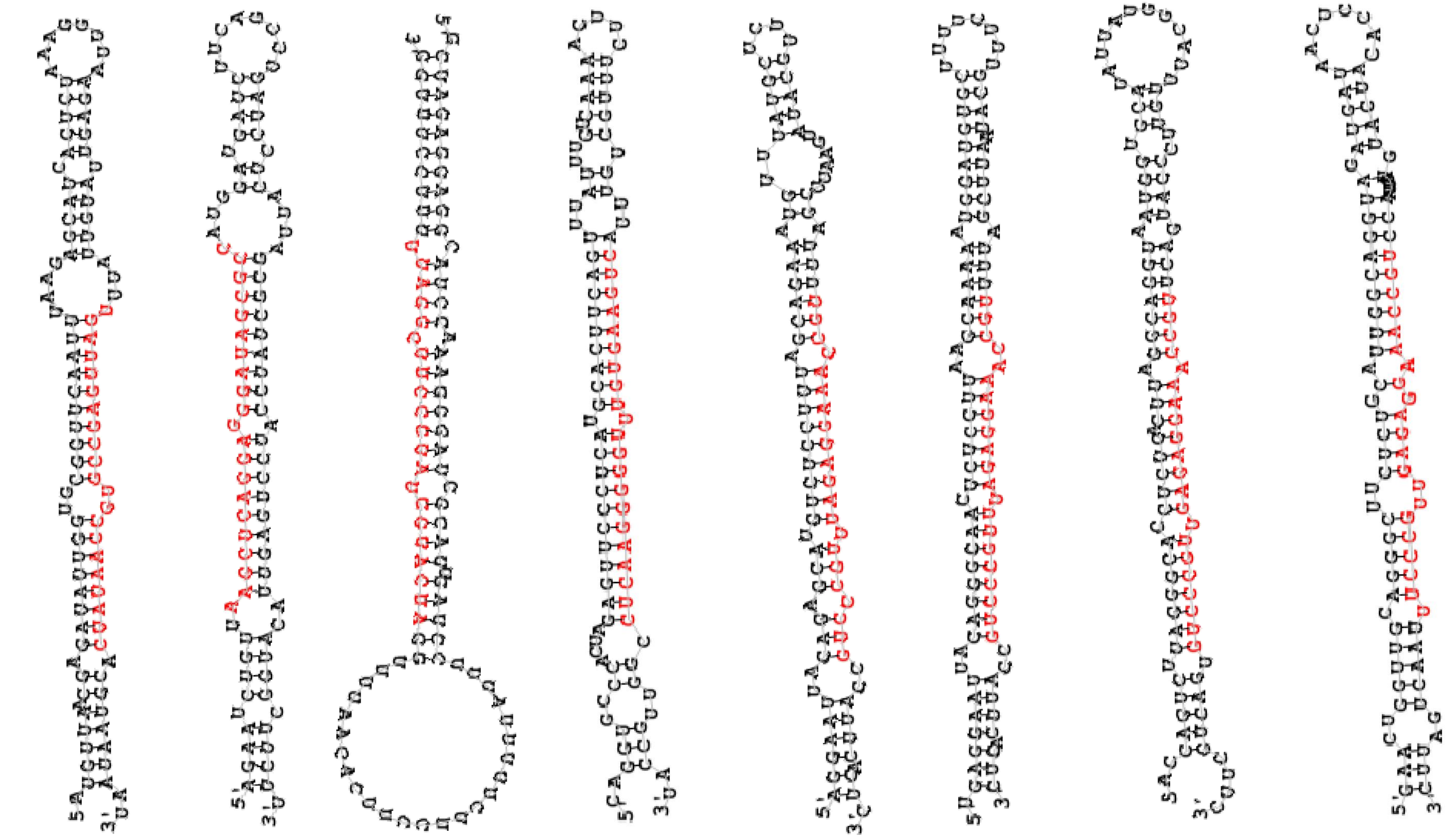

miR477a miR482-1 miR482-2 miR482-3 miR482-4 miR482-5 miR482-6

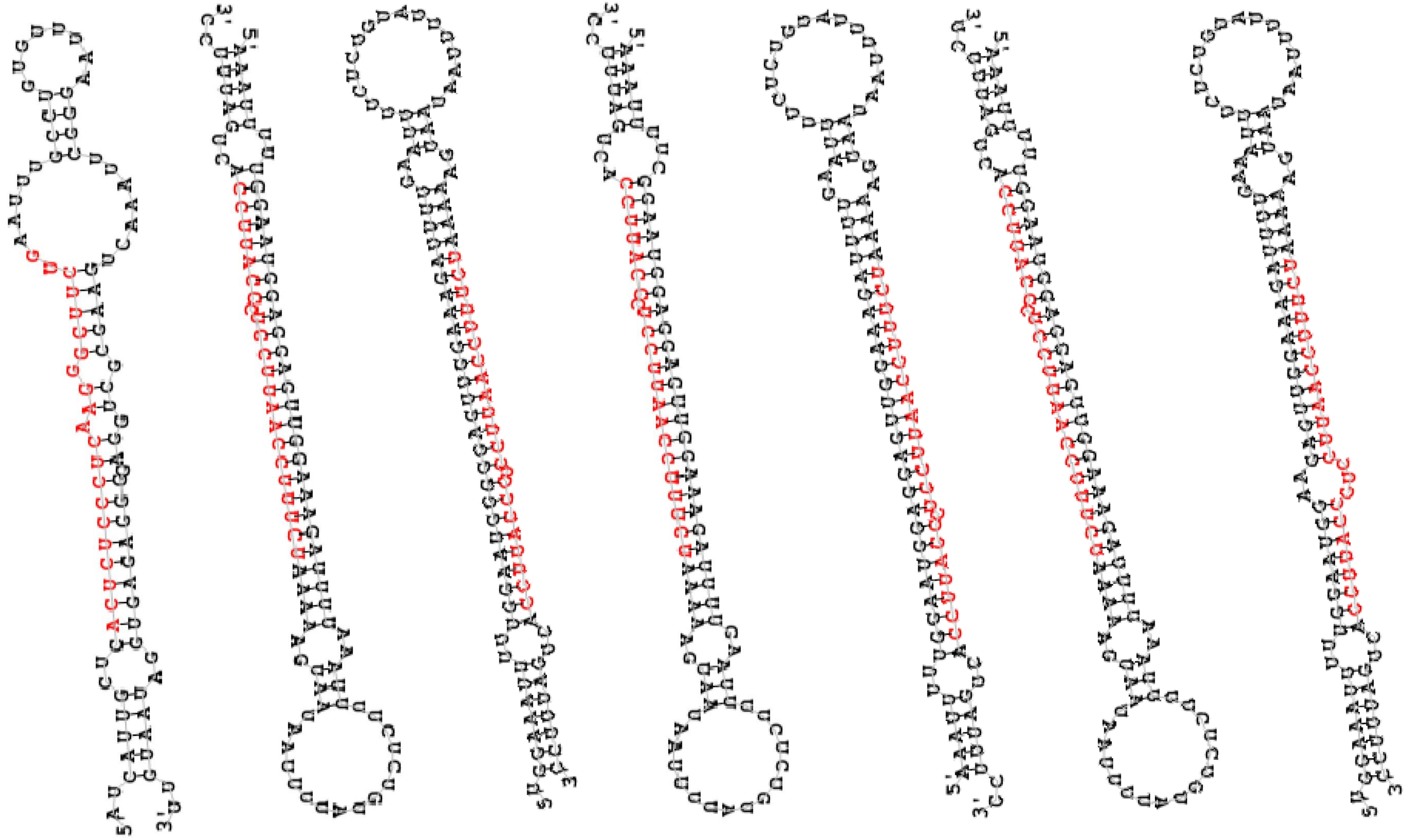

miR482b-3p-1 miR482b-3p-2 miR2111b miR2948-5p miR2950-5p-1 miR2950-5p-2

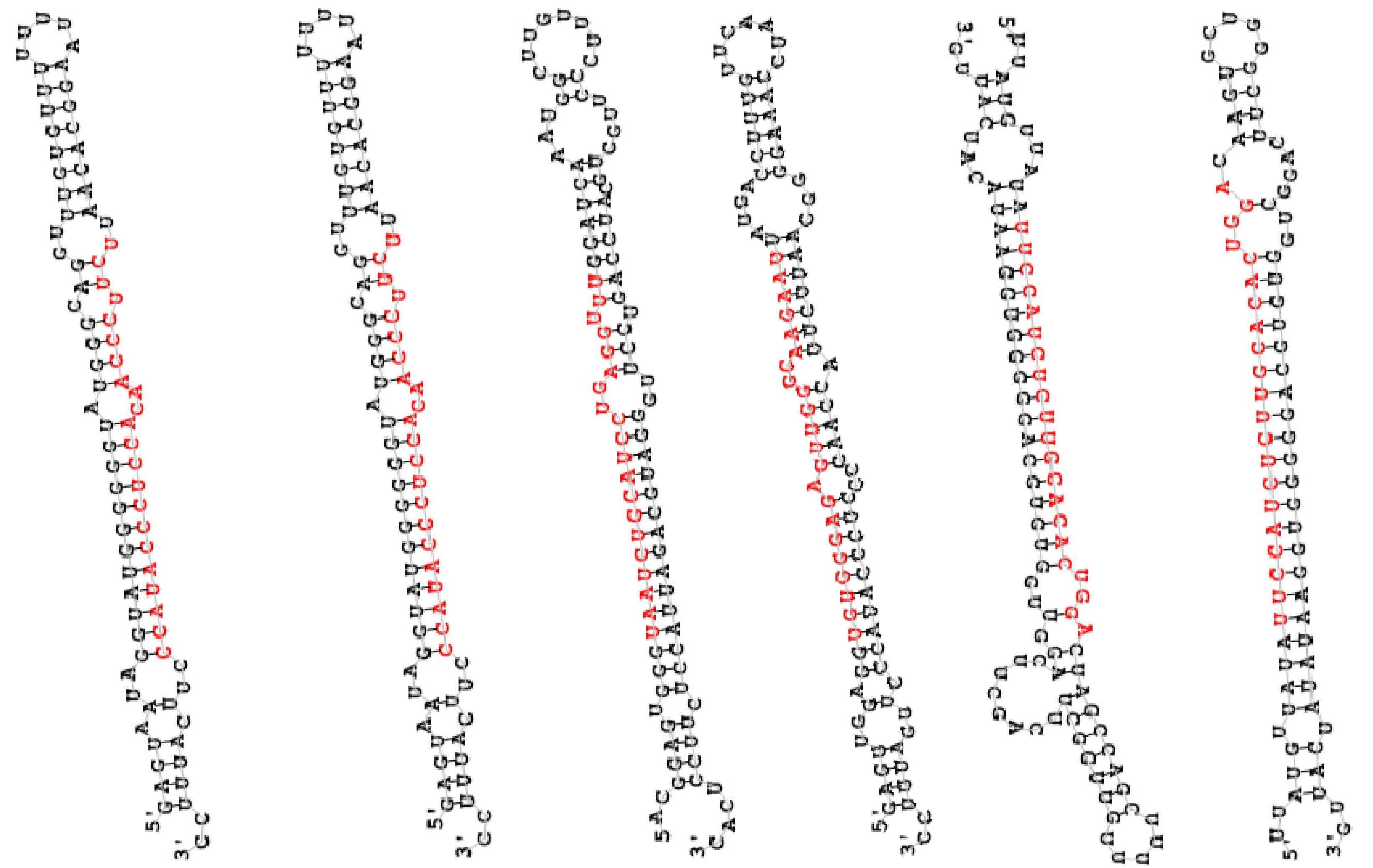

Supplement: Additional file 3: — Twenty nine precursors identified using miRDeep 2. (PDF 1319 kb) [file 12864_2015_2071_MOESM3_ESM.pdf]
