# Supplementary material for: MicroRNA and mRNA expression profiling analysis revealed the regulation of plant height in Gossypium hirsutum
Source: BMC Genomics. 2015 Oct 30;16:886. doi: 10.1186/s12864-015-2071-6 (PMC4628322; doi:10.1186/s12864-015-2071-6)
Supplement: Additional file 12: — The Illustration of small RNAs and target transcripts. Scatter plot diagrams show the frequency of tags and their positions on transcripts. The inferred cleavage sites were indicated by the Blocks and dotted line. (PDF 18 kb) [file 12864_2015_2071_MOESM12_ESM.pdf]

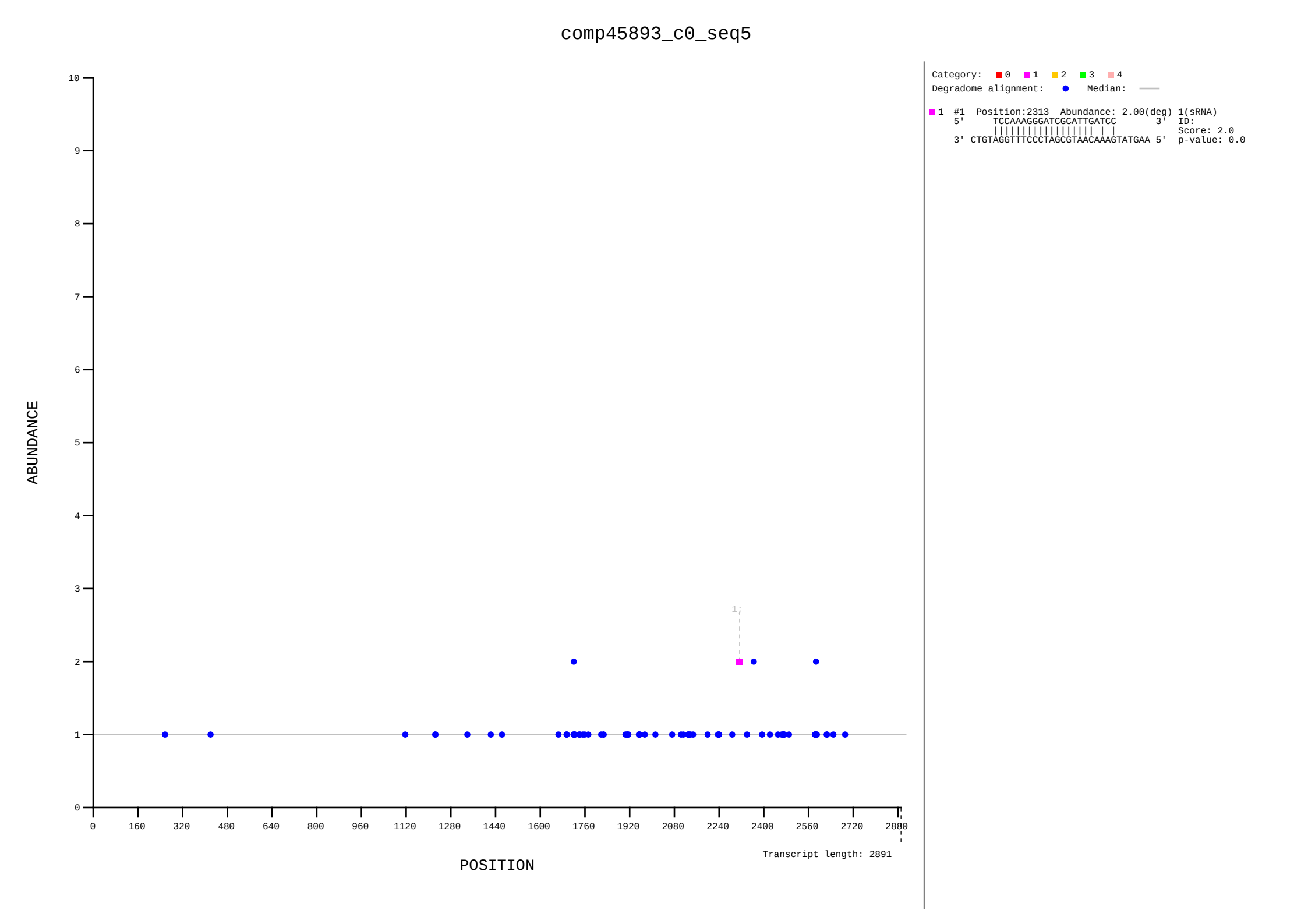
comp45893_c0_seq5
ABUNDANCE
POSITION
Transcript length: 2891
Category: 0 1 2 3 4
Degradome alignment:
Median:
1 #1 Position:2313 Abundance: 2.00(deg) 1(sRNA)
5' TCCAAAGGGATCGCATTGATCC 3' ID:
3' CTGTAGGTTTCCCTAGCGTAACAAAGTATGAA 5' Score: 2.0
p-value: 0.0

comp38451_c0_seq2

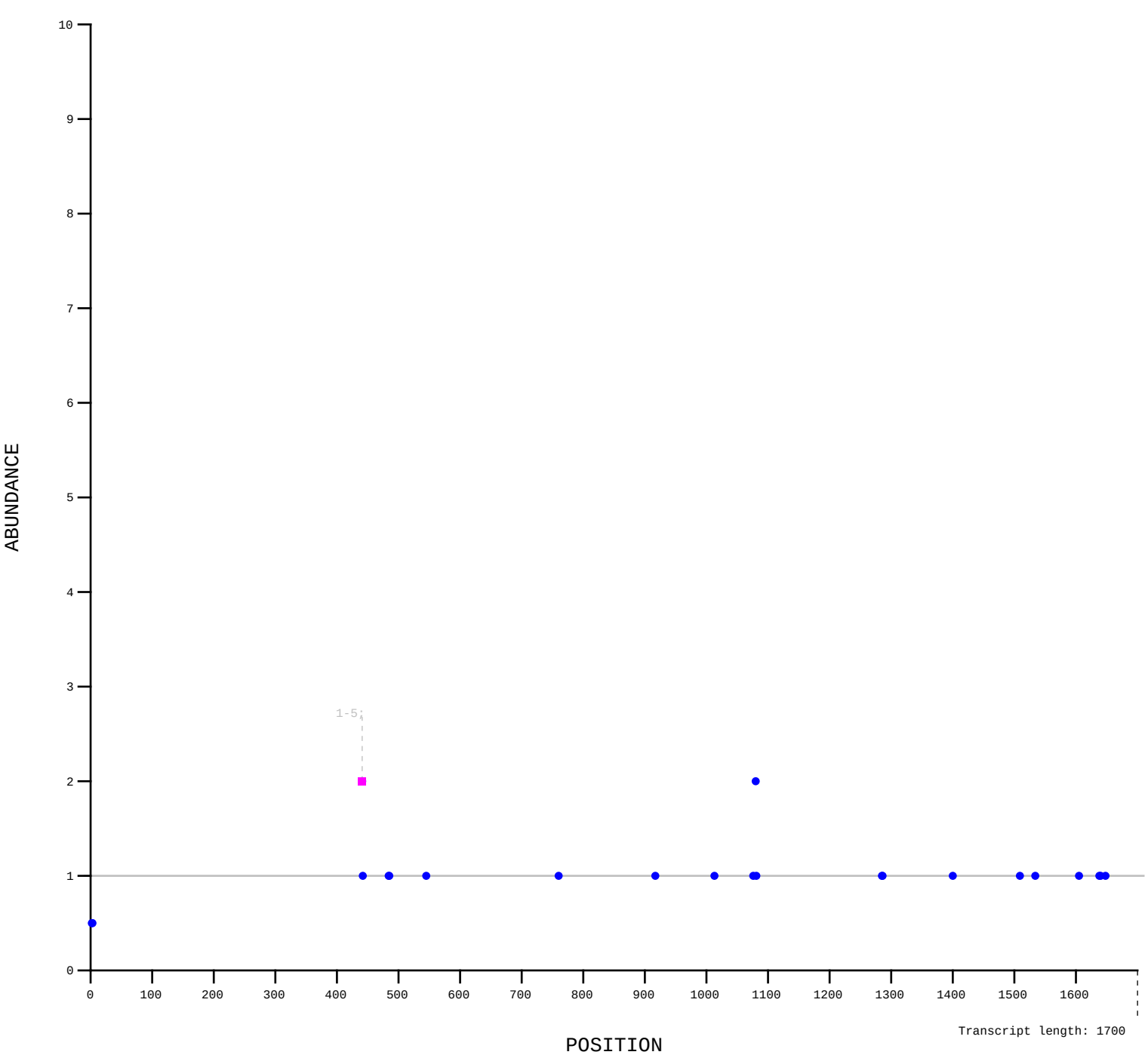

Category: 0 1 2 3 4
Degradome alignment: Median:

1 #1 Position:441 Abundance: 2.00(deg) 1(sRNA)
5' TGCCTGGCTCCCTGTATGCC 3' ID:
|||||||||||||o||||| Score: 0.5
3' TCGTACGGACCGAGGGACGTACGGTCGTCCTC 5' p-value: 0.0

1 #2 Position:441 Abundance: 2.00(deg) 1(sRNA)
5' TGCCTGGCTCCCTGTATGCCA 3' ID:
||||||||||||||o|||||| Score: 0.5
3' TCGTACGGACCGAGGGACGTACGGTCGTCCTC 5' p-value: 0.0

1 #3 Position:441 Abundance: 2.00(deg) 1(sRNA)
5' TGCCTGGCTCCCTGTATGCCG 3' ID:
|||||||||||||o|||||o Score: 1.0
3' TCGTACGGACCGAGGGACGTACGGTCGTCCTC 5' p-value: 0.0

1 #4 Position:441 Abundance: 2.00(deg) 1(sRNA)
5' TGCCTGGCTCCCTGAATGCCA 3' ID:
||||||||||||| |||||| Score: 1.0
3' TCGTACGGACCGAGGGACGTACGGTCGTCCTC 5' p-value: 0.0

1 #5 Position:441 Abundance: 2.00(deg) 1(sRNA)
5' TGCCTGGCTCCCTGAATGCCATC 3' ID:
||||||||||||| |||||| | Score: 2.0
3' TCGTACGGACCGAGGGACGTACGGTCGTCCTC 5' p-value: 0.0

comp45321_c0_seq12

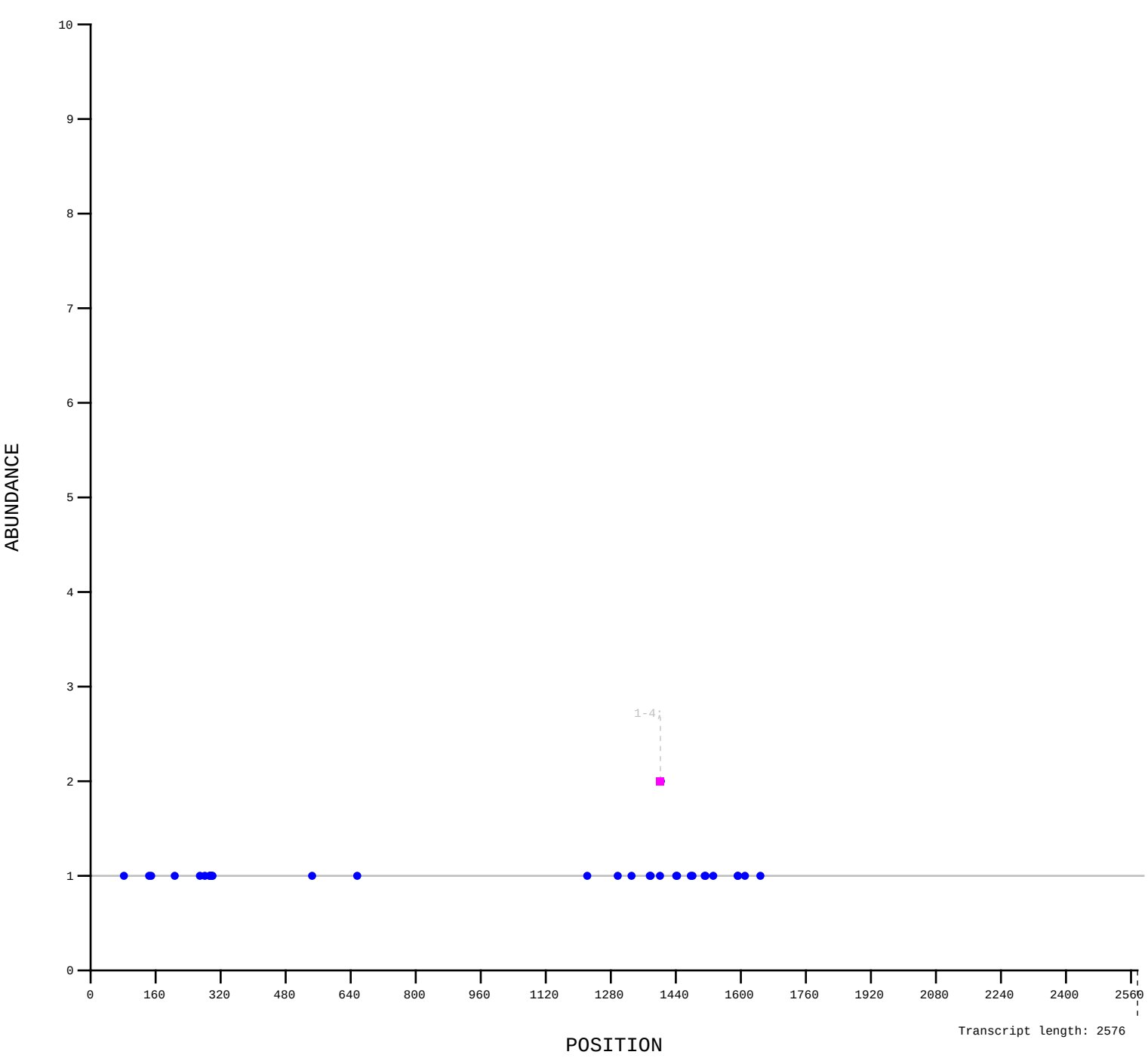

Category: 0 1 2 3 4
Degradome alignment: Median:

1 #1 Position:1402 Abundance: 2.00(deg) 1(sRNA)
5' AGAATCTTGATGATGCTGCA 3' ID:
|||||o||||||||||| Score: 0.5
3' ATCCTCTTAGGACTACTACGACGTCATCTCTT 5' p-value: 0.0

1 #2 Position:1402 Abundance: 2.00(deg) 1(sRNA)
5' AGAATCTTGATGATGCTGCAG 3' ID:
|||||o|||||||||||||| Score: 0.5
3' ATCCTCTTAGGACTACTACGACGTCATCTCTT 5' p-value: 0.0

1 #3 Position:1402 Abundance: 2.00(deg) 1(sRNA)
5' AGAATCTTGATGATGCTGCAT 3' ID:
|||||o|||||||||||||| Score: 1.5
3' ATCCTCTTAGGACTACTACGACGTCATCTCTT 5' p-value: 0.0

1 #4 Position:1402 Abundance: 2.00(deg) 1(sRNA)
5' TGAATCTTGATGATGCTGCAT 3' ID:
|||||o|||||||||||||| Score: 2.5
3' ATCCTCTTAGGACTACTACGACGTCATCTCTT 5' p-value: 0.01

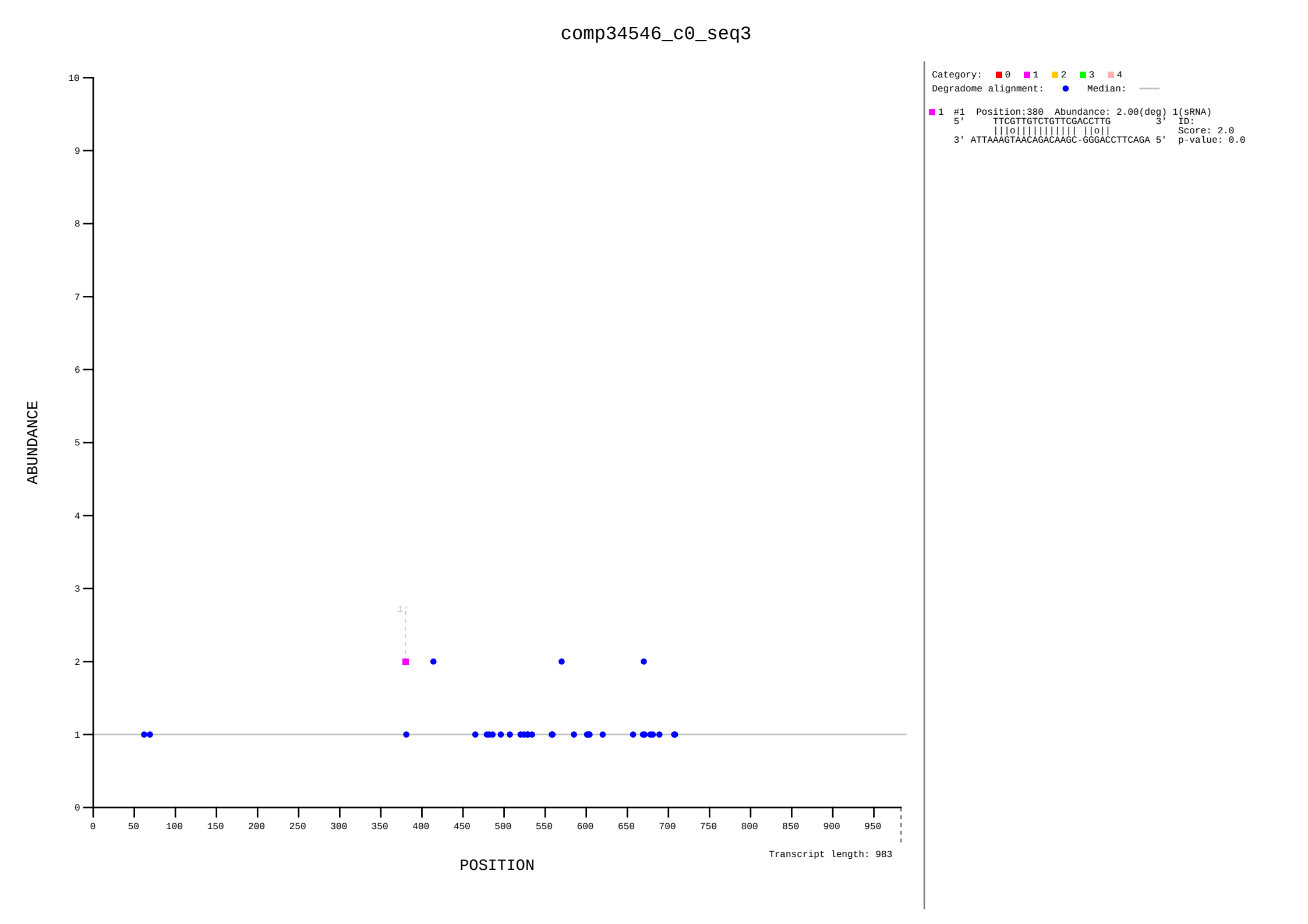
comp34546_c0_seq3
ABUNDANCE
POSITION
Transcript length: 983
Category: 0 1 2 3 4
Degradome alignment:
Median:
1 #1 Position:380 Abundance: 2.00(deg) 1(sRNA)
5' TTCGTTGTCTGTTCGACCTTG 3' ID:
Score: 2.0
3' ATTAAAGTAACAGACAAGC-GGGACCTTCAGA 5' p-value: 0.0

comp42588_c0_seq8

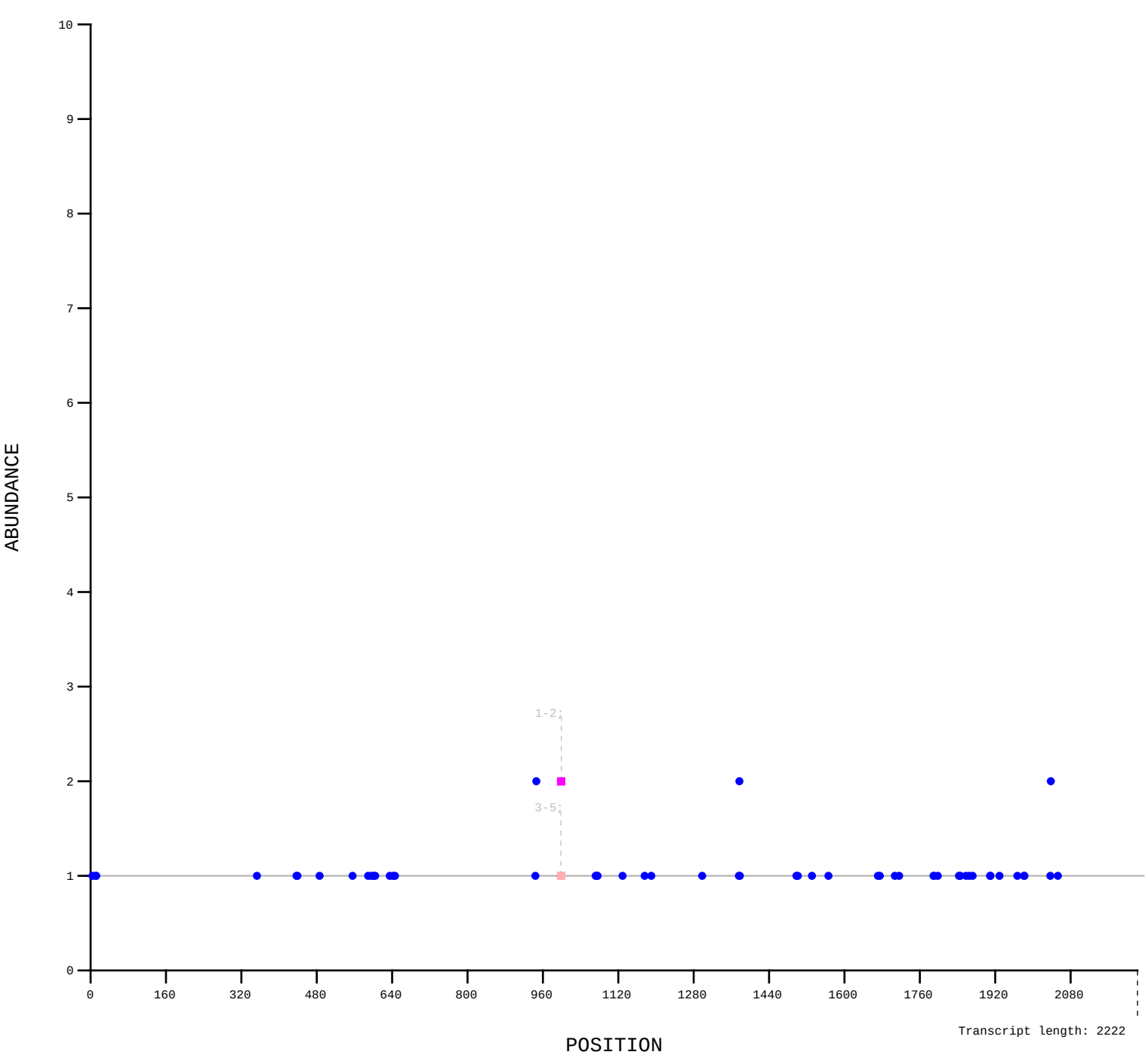

Category: 0 1 2 3 4
Degradome alignment: Median:

1 #1 Position:999 Abundance: 2.00(deg) 1(sRNA)
5' TTTGGACTGAAGGGAGCTCTA 3' ID:
|||||.|||||||||||o| Score: 2.5
3' CCTATAACCTCACTTCCCTCGAGGTTGAAGTG 5' p-value: 0.0

1 #2 Position:999 Abundance: 2.00(deg) 1(sRNA)
5' TTTGGATTGAAGGGAGCTCCT 3' ID:
|||||.|||||||||||| Score: 3.0
3' CCTATAACCTCACTTCCCTCGAGGTTGAAGTG 5' p-value: 0.01

4 #3 Position:998 Abundance: 1.00(deg) 1(sRNA)
5' TTGGATTGAAGGGAGCTCCA 3' ID:
|||||.|||||||||||||| Score: 1.0
3' CTATAACCTCACTTCCCTCGAGGTTGAAGTGT 5' p-value: 0.0

4 #4 Position:998 Abundance: 1.00(deg) 1(sRNA)
5' TTGGACTGAAGGGAGCTCCT 3' ID:
|||||.|||||||||||| Score: 2.0
3' CTATAACCTCACTTCCCTCGAGGTTGAAGTGT 5' p-value: 0.0

4 #5 Position:998 Abundance: 1.00(deg) 1(sRNA)
5' TTGGACTGAAGGGAGCTCCCT 3' ID:
|||||.|||||||||||| Score: 3.0
3' CTATAACCTCACTTCCCTCGAGGTTGAAGTGT 5' p-value: 0.01
